# Enhancing user-centred educational design: Developing personas of mathematics school students

**DOI:** 10.1016/j.heliyon.2024.e24173

**Published:** 2024-01-07

**Authors:** Robert Weinhandl, Martin Mayerhofer, Tony Houghton, Zsolt Lavicza, Lena Maria Kleinferchner, Branko Anđić, Michael Eichmair, Markus Hohenwarter

**Affiliations:** a Department for STEM Didactics, School of Education, Johannes Kepler University Linz, Altenberger Straße 69, 4040, Linz, Austria; b Department of Mathematics, Faculty of Mathematics, University of Vienna, Oskar-Morgenstern-Platz 1, 1090, Vienna, Austria; c Austrian Education Competence Centre Biology (AECC Biology), University of Vienna, Porzellangasse 4, 1090, Vienna, Austria

**Keywords:** Educational technologies, Mathematics student characteristics, Personas, Upper secondary education, UX research

## Abstract

Persona development techniques are a well-established method to create relatable descriptions of representatives of target users of digital systems. In the field of education, research on learner characteristics has yielded comprehensive results that can help advance educational approaches. Nonetheless, these results often remain abstract and distant for researchers and practitioners. Personas offer a bridge to make this knowledge more accessible and to facilitate user-centred design processes. This study focuses on creating personas of mathematics school students to ease such accessibility. These personas are constructed based on an understanding of learners’ goals, needs, challenges and problems, joys, fears, feelings and emotions, and strategies. Data collection was conducted through a multifaceted approach, encompassing qualitative and quantitative data from web surveys, think-aloud protocols, and interviews. The target demographic comprised upper secondary school mathematics students in Austria. We found five distinct patterns of characteristics prevalent in this target group. The patterns of characteristics reflected by the personas complement the scientific body of knowledge obtained from traditional approaches investigating characteristics and needs of learners. In practical terms, these personas empower the development of user-centred digital systems, learning materials, and lessons, thus fostering an enriched educational experience for mathematics students.

## 1 Introduction

Information and communication technologies are changing how mathematics is being taught and learned in schools [1,2]. In this paper, we consider subject-independent technologies such as learning management systems as well as subject-specific technologies, for example, dynamic geometry software and computer algebra systems. The use of such technologies is required by mathematics curricula in Austria [3] and further educational policy recommendations [4]. Nevertheless, the impact of introducing new technologies on teaching and learning mathematics is complex [1]. According to Sinclair [2], new technologies are not only changing how mathematics is taught and learned, but also impacting the mathematical content itself. In view of the complex interplay among content, teachers, and learners, it is important to identify typical characteristics and needs (e.g., engaging, non-participating, structured, disorganised) of mathematics learners and their interactions (e.g., non-participating and structured) in order to offer them suitable learning environments, i.e., the physical settings in which learning takes place including the technological infrastructure.

Personas are an established tool to incorporate the characteristics and needs of potential users of digital systems. Personas are archetypical, fictional users of a product or service [5] that capture, for example, potential users’ goals, needs, challenges, and emotions [[6], [7], [8], [9], [10]]. Persona development techniques have been widely utilised in user interface design and in user experience research (UX research) as a tool to qualitatively study and describe potential users of a product or service [5]. The central objective of UX research is to understand potential users’ needs and their characteristics in relation to the aimed usage [11]. Based on the literature, UX is broadly viewed as an individual experience “that arises from the interaction with a product, system, service, or an object” and as a “dynamic, context-dependent and subjective” concept [12]. Experts point to the following benefits of the utilisation of personas to support the development of digital resources: (1) Personas can improve developers’ understanding of the needs and preferences of the target group and assist them designing digital resources that may be better attuned to these needs and preferences [13]; even though in different contexts, empirical evidence has been found that the use of personas can increase the accuracy of preconceptions about the target group represented by the personas [14]; (2) personas can be used to argue about the design decisions, facilitate communication about designs, and foster creativity and engagement in the design processes [13,15]; (3) personas can help researchers to communicate their research findings and inform changes in educational policies [16]. Therefore, personas can be considered as valuable tools for user-centred approaches in education and for the development of digital learning resources.

Persona development has been primarily employed in software development, healthcare, and higher education [17], but their potential in secondary education is essentially untapped. At the same time, the use of technology in secondary education has become compulsory in numerous countries and the integration of technologies into STEM and mathematics has been accelerating during the past years, particularly boosted by the COVID-19 pandemic. Thus, personas of school students can be a valuable tool to support design processes.

In contrast to product design, where the goal is to create attractive products and to increase the purchase intention of the consumers [18], educational technologies aim to support learning processes. The “products” that our study addresses are digital mathematics learning resources or environments that users interact with; the “users” in our case are upper secondary school mathematics students in Austria. By creating personas of upper secondary school mathematics students, our study aims to support the development of digital mathematics learning resources that contribute to optimising learning outcomes. For this reason, the goal of the present study is to answer the following research question:

What are the dominant patterns of characteristics of upper secondary school mathematics students in Austria?

In the development of digital learning tools, the characteristics of the learners are important. Research based on psychological measures of student characteristics shows that it can be difficult to translate research findings into actual school practices. Our aim is that the personas developed in this project contribute to research by reflecting typical patterns of characteristics of mathematics learners which serve as a supplement to other available characterisations of students that are based on approaches from educational psychology such as motivational profiles of school students [19] or clusters of STEM students based on motivational trajectories [20]. We intend that the personas can bridge findings from research with design processes in practice and be of practical use for developers of digital resources for teaching and learning mathematics as well as for teachers in lesson design.

## 2 Theoretical background and literature review

The use of technologies has become mandatory in the Austrian secondary school leaving examination in mathematics [3]. This change mandates the use of technologies such as *GeoGebra* or *TI-Nspire* throughout upper secondary education to support teaching and learning. The COVID-19 pandemic, the associated distance learning phases, and the period after school closures revealed that, in addition to mathematics-specific software, the use of mathematics-independent digital tools such as learning platforms or learning videos can also improve mathematics learning [21]. Developing mathematics learning resources using technologies for teaching and learning mathematics requires a sound understanding of the characteristics and needs of upper secondary school mathematics students. As stated earlier, the principal aim of our study is to identify characteristics of Austrian upper secondary school mathematics students and the interaction of such characteristics within their technology-rich environments. To explain our choice of research approaches, we first review the approaches of related studies.

### 2.1 The role of student characteristics in learning mathematics

In the 1960s, researchers started to investigate relationships between teaching methods and learner characteristics systematically. The assumption of the existence of *aptitude-treatment interaction* (ATI) started to evolve [22]. ATI proposes that there are optimal teaching methods (= treatment) with regard to the learning progress specific to the particular characteristics of learners (= aptitude). However, neither the study of Tallmadge [23] on the relationship between aptitude and teaching methods nor extensive literature reviews of Bracht [24] and Hayes and Allinson [25] on this topic have substantiated this assumption. Hunt [26] suggested that the lack of significant results in researching ATI may be related to the overwhelming focus on quantitative methods. Accordingly, researchers may be relying too heavily on quantitative measures and statistical analyses to study the relationship between teaching methods and learner characteristics, which speaks in favour of including qualitative data.

The importance of individual student characteristics is stressed by the Austrian curriculum for academic upper secondary schools [3], which requires teachers to align their teaching with the diversity of their students in terms of previous experience, culture, language, and gender. This policy has supported the development of pedagogical approaches that promote individualised teaching and learning. Such pedagogical approaches shape mathematical learning environments with the goal of improving the proficiency of mathematics learners. Today, mathematical learning settings often include technologies as they provide students with affordances to individually choose the level and to proceed at an individual pace. Designing learning environments and resources that accommodate the individual demands of learners is still a challenge for teachers and developers of learning resources. To render design processes of learning resources and settings more learner-centred, our study aims to make the characteristics and demands of learners more easily accessible and relatable, thereby supporting those who develop digital mathematics learning resources to take into account the characteristics of a wide range of potential users. Therefore, we develop concrete descriptions of mathematics learners that are representative of students in actual classrooms. To develop such representative descriptions of mathematics students in academic upper secondary schools in Austria, we use persona development techniques. The purpose of developing personas in this context is to create descriptions of prototypical mathematics students, to help empathise with prototypical mathematics students in the design processes of digital mathematics learning resources, and to provide a new student-centred research approach in mathematics education research. While this new approach to mathematics education research represents an important addition to the field, it should be considered a supplement to existing research frameworks such as learners’ goal orientations [27] and person-oriented approaches that aim to cluster students according to specific characteristics [19].

### 2.2 Using technologies in teaching and learning mathematics

The use of technologies in teaching and learning mathematics has gained importance during the past years. In Austrian academic upper secondary schools, this development has been promoted by policy changes. The use of technologies has been a fundamental curricular principle since 2017. The current curriculum requires the use of technologies to, for example, manipulate terms, compute derivatives and anti-derivatives, solve equations and compute integrals numerically, or plot functions at least occasionally.^1^ Accordingly, the compulsory written school leaving exam in Austrian academic upper secondary schools encompasses items that require the use of technologies.^2^ The COVID-19 pandemic has propelled the use of technologies in schools (e.g., Refs. [21,29]). To develop digital mathematics learning resources and choose appropriate technologies, it is necessary to understand characteristics and demands of students in Austrian academic upper secondary schools.

According to Larkin and Milford [30], the selection of suitable tools for teaching mathematics from a wide range of possibilities is a particular challenge for teachers. The rapid emergence of new technologies in adolescents’ daily lives has increased the pressure on schools to integrate technologies into teaching [30]. International organisations such as the European Union and the Organisation for Economic Co-operation and Development (OECD) have declared the use of technologies an educational goal, also adding to this pressure [4]. The role of teachers is crucial for the effective use of technologies in mathematics classrooms. The established framework of *Technological Pedagogical Content Knowledge* (TPCK/TPACK) highlights the relevance of various dimensions of teacher knowledge for successful implementation of technologies into teaching and learning mathematics. According to this framework, technological knowledge, knowledge of mathematical content, and pedagogical knowledge as well as knowledge about the interplay of these knowledge areas are required from teachers in teaching mathematics with technologies [31].

According to Donevska-Todorova and Trgalova [1], integrating technologies into learning mathematics is highly complex. Their use changes both how learning happens and what is learned [2]. Calder and Murphy [32] explain that using apps, i.e., software applications designed to run on mobile devices or computers, has the potential to change the mathematical understanding and associated mathematical thinking of learners. Furthermore, according to numerous studies and meta-studies, the use of technologies in teaching and learning mathematics has the potential to foster collaboration among students [33] and to render the learning process more enjoyable and engaging [34]. In order to optimise these technologies, they should be adapted to the characteristics of the students.

Despite the growing popularity of digital educational technologies and policies that prescribe their implementation in learning, there is a significant lack of research on how well these technologies are adapted to learners’ needs and characteristics [35]. Schmidt and Tawfik [36] indicate that the application of persona development techniques can contribute to reducing the gap in knowledge about the approaches and strategies used by students while working with digital technologies, as well as to reducing the lack of approaches for technology developers or teachers to consider the characteristics of students.

### 2.3 Personas

According to Cooper [5], personas are “hypothetical archetypes of actual users” of a system that are developed in a design context and based on the goals of the users. Anvari et al. [37] have proposed *holistic personas* which focus on *factual*, *personality*, *intelligence*, *knowledge*, and *cognitive process* dimensions when creating personas. These holistic personas represent an abstract artefact with generic properties and attributes. For the purpose of developing digital technologies and digital learning settings, Lilley et al. [6] and van Rooij [10] focus on the demands, desires, experiences, and challenges of potential users when creating personas. In line with Liston and O’Donoghue [38] and Roesken et al. [39], key dimensions of mathematics students include abilities, mathematics difficulties, enjoyment of mathematics, mathematical self-concept, value of mathematics, and success. These dimensions of personas are related to the Big Five personality traits, which are Factor I, *Surgency* (or *Extraversion*); Factor II, *Agreeableness*; Factor III, *Conscientiousness*; Factor IV, *Emotional Stability* (vs. *Neuroticism*); and Factor V, *Culture* or *Intellect* [40]. These factors find expression in specific behaviours, for example, activity or passivity (Factor I); trust or distrust (Factor II); organisation or carelessness (Factor III); imagination, curiosity, and creativity or perceptiveness and shallowness (Factor V). In this way, four of the Big Five personality traits are connected to the structure and the dimensions of personas of mathematics learners.

Personas were originally developed to render software development processes more effective in meeting the needs of target users by making the target users more relatable for developers [41]. Using personas makes it easier for developers to empathise with user groups and reduce preconceptions that might influence their decisions. Personas are useful for designing digital tools in educational contexts, facilitating understanding of student demands, and making implicit teacher knowledge visible [6,16]. Personas represent the members of the target group of a system and form a synthesis of information on the goals, motivations, and skills of a subgroup of the target population [5]. Personas provide an “imperfect but compelling lens” on users and user demands [42]. A persona is often given by name, picture, personal background, and, most importantly, a description of relevant characteristics and demands [6,7,10].

The scientific literature distinguishes two ways in which personas are presented, namely *dashboard personas* and *narrative personas*. According to Minichiello et al. [16], dashboard personas present the information on the individual personas of a population in the form of a table and include short summaries of their personal background and relevant details. Narrative personas present information entirely in prose. Furthermore, personas are categorised with regard to the kind of data that has been included in their development. There are *ad-hoc personas* that are developed based on personal assumptions and *data-driven personas* that are developed based on systematically collected data [16].

Regarding the development of learning and learning environments, personas have already been used to adapt the design of distance learning environments to students’ demands [6]. Focusing on STEM subjects, for example, personas were used to redesign an online data visualisation platform for university teachers [43].

### 2.4 Potential shortcomings of personas

Persona development has been criticised for being too subjective [10,41,44] and according to Ferreira et al. [44], data used in the development of personas are frequently “informal and unscientific”. To reduce the degree of subjectivity in persona development, we collected data in line with predetermined procedures, questions, and tasks.

Another criticism of persona development processes is that not all potential users of a system are represented in the data used, that data samples used in the process include users that are not part of the target group of a system, and that the number of data samples used in the process is often too small [43,45]. This may lead to superficial, unauthentic personas which do not realise the potential of the technique.

## 3 Methods

### 3.1 Data for persona development

According to a range of researchers [16,41,43], persona development should be based on data that is both extensive and rich. It is recommended that both qualitative and quantitative data [45] as well as primary and secondary data [42] are incorporated. In this context, *primary data* means that data are collected directly from the potential users of a system; data collected about potential users are referred to as *secondary data* [42]. Although any information about potential users could be used in persona development, Minichiello et al. [16] and Zaugg and Rackham [9] report that mainly qualitative data have been used. Thus, we included both quantitative and qualitative data in our study. The qualitative secondary data were collected from mathematics in-service teachers at academic upper secondary schools and from mathematics pre-service teachers. In addition to in-service teachers, we incorporated pre-service teachers at the end of their bachelor’s degree programme as they are closer in age to upper secondary school mathematics students and closer to being school students themselves. This suggests that they still have a very lively knowledge of learning mathematics from the student perspective. Also, the pre-service teachers have already completed extensive content and pedagogical-didactic university courses, which suggests that they have acquired basic mathematics knowledge for teaching. This mix of non-formal and formal knowledge makes pre-service teachers a valuable source of expertise for our study [46,47]. We used different tools to collect comprehensive data regarding the development of personas.

According to previous research [9,10,41,45,48], persona development involves collecting both qualitative and quantitative data; moreover, interviews and observations are commonly used to collect qualitative data about existing and potential users, while web surveys can help expand the geographical range of respondents. To include quantitative data, primary or secondary data can be collected; alternatively, previously collected data from databases can be used. In our study, we used web surveys to collect secondary data about academic upper secondary school mathematics students as a first step. Based on this data, persona prototypes were developed.

**Fig. 1:**
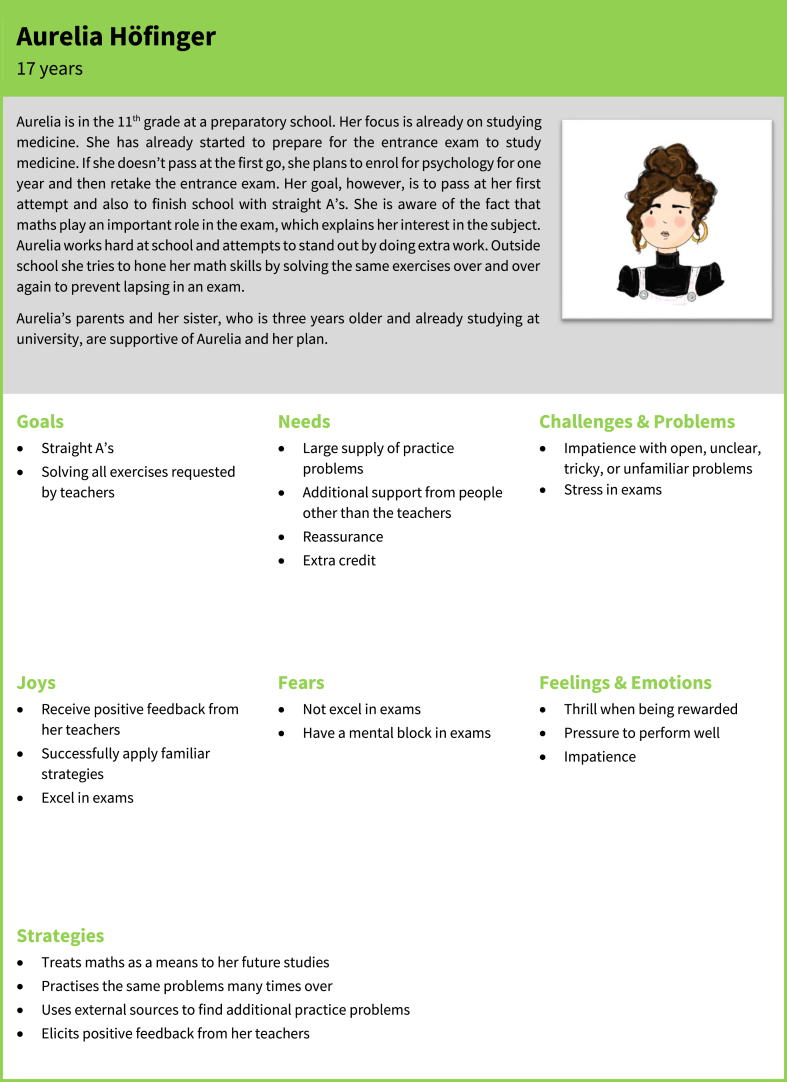
Example of a persona in dashboard form from our study.

In this study, we used persona development techniques to create a structured representation of student needs related to technology. Specifically, we created data-driven dashboard personas (see Fig. 1). These personas should guide the development of learning environments and educational technologies. The personas developed in this study provide a first systematic record of characteristics and combinations of characteristics of the student population. Certainly, they may be refined as additional data becomes available in future studies.

**Fig. 2:**
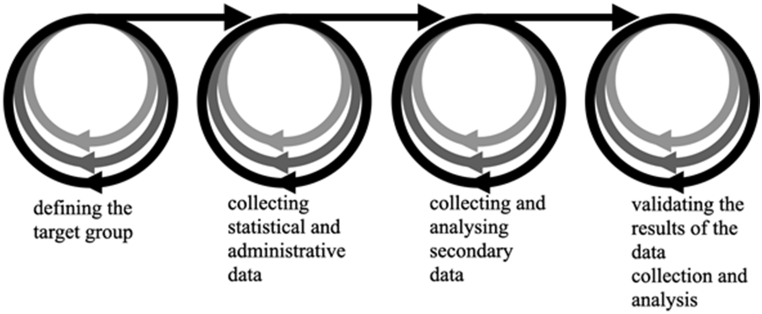
Steps and one sequence of developing personas.

Our process of developing personas consists of the following four steps (see Fig. 2): (1) definition of the target group for developing personas; (2) collection of statistical and administrative data; (3) collection and analysis of secondary data; (4) validation and improvement of the resulting personas. These four steps were carried out in a linear sequence; each individual step was iterated.

### 3.2 Target group

The target group of our study are academic upper secondary school mathematics students. These are students from the ninth to the twelfth grade, mostly between 14 and 18 years old. They were selected as the target group because mathematics is a compulsory part of their written school leaving exam and because of specific regulations [28] for this exam that mandate the use of technologies.

### 3.3 Statistical and administrative data

Statistical and administrative data on the mathematics students in the target group and on their teachers were taken from *Statistics Austria*^3^ and the National Education Reports 2015 [49] and 2018 [50]. These data framed our data collection strategy and were used while developing the individual personas. In the school year 2019/20, there were 352 academic upper secondary schools [51]. In the school year 2020/21, more than 87,000 students attended academic upper secondary schools [52]. According to the National Education Reports 2015 [49] and 2018 [50], around 60 % of the students and 60 % of the teachers in academic upper secondary schools in Austria were female; there are roughly as many students attending these schools in urban and in nonurban areas.^4^ Notably, Vienna accounted for almost 30 % of the total student population in academic upper secondary schools. The participants were selected based on gender and geographical aspects in the administrative data, with slightly fewer than a quarter of the participants from schools in Vienna.

### 3.4 Questionnaire development

In our study, we used qualitative web surveys to collect secondary data to extend the geographical scope and thus better capture the population of in-service and pre-service mathematics teachers. Previous research suggests the use of open-ended qualitative surveys to collect data for persona development [6,42]. According to Nielsen [53], the design process of tools for professional use may benefit from personas that focus not only on the desired outcome of work processes but also on the attitudes and emotions of the target users. We include these recommendations in the design of our questionnaire which addresses the categories *goals*, *needs*, *feelings & emotions*, *joys*, *fears*, and *strategies* (see Table 1). As our study is carried out with school students, we refer to strategies rather than to “work procedures”. Following Lilley et al. [6] and van Rooij [10], we also include *challenges & problems*. When selecting these aspects, we also took into account the dimensions and factors described in section 2.3.

**Table 1:** Central themes of the online questionnaire.

Themes	Specific themes or questions
A: Goals	(A1) goals that the student pursues in or through their mathematics education; (A2) challenges that the student faces in achieving their goals
B: Needs	(B1) What are the student’s needs in mathematics education? (B2) How can the student be supported in meeting those needs? (B3) What means does the student use to meet those needs?
C: Challenges & Problems	(C1) What challenges/problems does the student face when learning mathematics? (C2) What strategies does the student use to overcome these challenges/problems?
D: Joys	(D1) What does the student enjoy most about learning mathematics? (D2) What means are there to maintain or increase this enjoyment of learning mathematics?
E: Fears	(E1) What is the student most afraid of in mathematics lessons or when learning mathematics? (E2) What can be done to reduce this fear in mathematics lessons or when learning mathematics?
F: Feelings & Emotions	(F1) What emotions does the student display when learning mathematics? (F2) How do these emotions manifest themselves?

Cooper [5] recommends that researchers or experts develop descriptions of potential users rather than involving potential users themselves. These descriptions should then guide the design decisions of the developers—in our case decisions on the design of digital tools for teaching and learning mathematics. Although the questionnaire is not directly linked to technology development, the personas that emerge are specifically designed to aid in the creation of technology-enhanced learning resources and environments. As such, the questionnaire serves as a valuable tool for generating user profiles that can inform the design process and enhance the development of user-centred educational technologies.

In the first part of our qualitative web survey, the participants were asked to choose and focus on one particular mathematics student who they consider typical. Participants were then asked to provide general information about this student and to share their personal evaluation of the student’s talent for mathematics, of how much the student enjoyed mathematics, and whether or not mathematics seemed likely to play an important role in the later education or profession of the student. In the second part, questions on the presumptive goals, needs, challenges and problems, joys, fears, feelings and emotions, and strategies of the student that they have in mind were asked. The central themes and questions in the secondary data collection are shown in Table 1. Recognising that there is no *typical* mathematics school student, we employed a qualitative web survey to increase the diversity of participants in our data collection. By using this method, we were able to gather a broad range of perspectives and experiences to enhance the richness and representativeness of our findings. To explore the common combinations of characteristics of mathematics students, we utilised an age and experience dispersion approach in addition to the geographical dispersion. Thus, we collected data on academic upper secondary school mathematics students from both in-service mathematics teachers and pre-service teachers using a web survey. The wide range of participants in our study in terms of age, gender, teaching experience, or whether participants come from rural or urban areas, should contribute to providing diverse perspectives on academic upper secondary school mathematics students in Austria. These diverse perspectives enabled us to use the data provided by the participants and the persona techniques to develop accurate descriptions of upper secondary school mathematics students in Austria and their characteristics and demands.

For the data analysis, only the questionnaires whose first part had been filled in completely were used. In the following section, we explain in detail how the data collection was carried out.

### 3.5 Data collection

In our study, qualitative secondary data on academic upper secondary school mathematics students in Austria were collected in addition to quantitative data (see section 3.2). Following the recommendations of Ferreira et al. [44], we utilised guiding questions to set up a systematic way of collecting data for our study. We collected secondary data on the themes presented in Table 1. The questionnaire was sent to in-service mathematics teachers and to pre-service teachers in the authors’ networks, all while observing the socio-demographic distributions described in Section 3.2. The in-service teachers were asked to describe one specific student who they considered typical. The pre-service teachers were invited to complete the questionnaire while thinking of themselves as a student and to complete a second questionnaire filling in the characteristics of a former fellow student who had not been good at or had not liked mathematics in upper secondary school. This is to ensure that both students who had enjoyed or had not been good at mathematics and students who had not enjoyed mathematics at school were included in the data collection procedure. In this study, we did not collect primary data.

The selection of the schools to recruit teachers from was made according to the distribution of mathematics teachers in Austrian academic upper secondary schools: A nearly equal number of in-service mathematics teachers and pre-service mathematics teachers from urban and nonurban regions with Vienna as a core area were included in our study. 13 in-service mathematics teachers and 61 pre-service mathematics teachers completed the online questionnaire. Of these 74 qualitative web surveys, the level of completeness of 47 was such that they could be used for further analysis. To guarantee full anonymity and to increase the willingness of teachers to participate in our study, neither age nor gender were asked. Details on the participants and the collected data are presented in Table 2.

**Table 2:** Participants and sources of data used to develop the personas.

Participants	Sample size	Instrument
in-service mathematics teachers in upper secondary schools	n = 7	qualitative questionnaire
pre-service mathematics teachers	n = 40	qualitative questionnaire
upper secondary school students	whole population	public statistical databases

### 3.6 Analysis of the secondary data and development of the personas

**Fig. 3:**
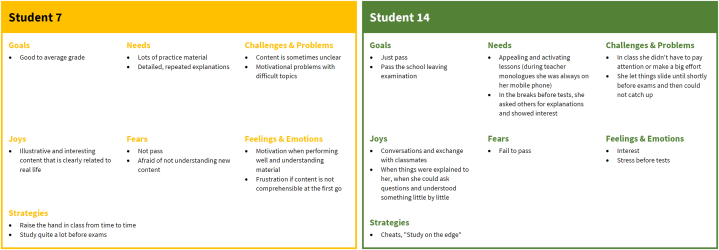
Exemplary responses from the qualitative web surveys; entire dataset available as Multimedia component 1.

To develop personas, we processed the data in four steps. The responses of each participant were entered into a table and translated from German to English by the authors (see Fig. 3). The result is attached to this paper as Multimedia component 1. Each table contains a brief verbal description of the student as well as information on their characteristics with regard to the seven categories *goals*, *needs*, *challenges and problems*, *joys*, *fears*, *feelings and emotions*, and *strategies* as reported by the respective respondent. These data were processed following these four steps:

*Step 1: Creating codes and clustering student descriptions.* In this step, the first and the second author individually analysed the tables. They created codes inductively and clustered the tables by similarities in the codes. To these initial persona prototypes, the authors each assigned a keyword and a brief description.

Step 2: *Clustering initial persona prototypes*. With the aim to increase the level of abstraction, each researcher individually compared and merged the initial persona prototypes. In this process, the initial prototypes were grouped in terms of the verbal descriptions and the information in the seven persona categories. New persona prototypes of a higher level of abstraction were intended to result from this step.

*Steps 3 and 4: Condensing persona prototypes.* In these steps, the process from Step 2 was repeated jointly by the first and second authors of this work to increase the level of abstraction in the personas and to reduce the total number of personas. In the fourth step, the persona prototypes were attempted to be further condensed. Based on administrative and statistical data, additional information such as gender, migration history, school district (rural/urban), or socio-economic background was added to the personas. Additional information such as family background, general hobbies, and particularly relevant extracurricular activities was added to the respective personas based on both the secondary data and the administrative and statistical data.

### 3.7 Validating and improving the personas

The personas developed in the way described above were validated (see overview in Table 3). In the validation process, the personas were presented to 14 in-service mathematics teachers in academic upper secondary schools who had not been involved in the initial data collection process. They were asked to check whether they could relate the individual personas to actual mathematics students they had been teaching. Moreover, they were asked to add, refine, or delete any information in the individual personas that appeared to be off to them. The feedback was collected face-to-face or online from twelve teachers and then used to adapt the personas accordingly. Afterwards, the personas were presented to 83 academic upper secondary school students and the students were asked to improve the personas by adding or deleting information in the provided personas.

**Table 3:** Participants and sources of data used to improve the personas.

Participants	Sample size	Instrument
in-service mathematics teachers in upper secondary schools	n = 14	think aloud protocols
in-service mathematics teachers in upper secondary schools	n = 4	quasi-unstructured interviews
university faculty members	n = 3	quasi-unstructured interviews
pre-service mathematics teachers	n = 6	semi-structured interviews
upper secondary school students	n = 83	written feedback

Finally, the personas were used in practice, namely in the project FLINK. FLINK is a project in which pre-service mathematics teachers develop open digital mathematical learning resources with the support of in-service mathematics teachers and university staff. To capture their experiences in the use of the personas, quasi-unstructured interviews were conducted with a particular focus on *gaining trust* and *establishing rapport* [54]. Both the pre-service and the in-service mathematics teachers reported back on what aspects of the personas they found to be particularly helpful for their work and what they considered irritating or dispensable. Six pre-service mathematics teachers (female: n = 6), four in-service mathematics teachers (female: n = 2; male: n = 2), and three academic staff members (female: n = 2; male: n = 1) provided feedback on the personas, which they had all used for their work. The in-service teachers had 10–20 years of teaching experience; the university staff members were all teaching in the field of mathematics education.

Using the techniques described above to improve the personas, a sufficient fit of the personas was reached.

The personas of academic upper secondary mathematics students in Austria resulting from this process are described in the following section. The dashboards are attached to this work as Multimedia component 5 and can be viewed in the German original and in English.

The personas resulting from our systematic study may now serve as a basis for future research. The incremental nature of persona development is a feature rather than a fault [9,42,48]. The target group is constantly changing and personas have to be revised continuously to accurately reflect the characteristics of potential users.

## 4 Results

The collection of data and their analysis resulted in five personas. We present the intermediate results after each step described in section 3.6 as well as the results of the validation process below.

### 4.1 Development and validation of personas

**Fig. 4:**
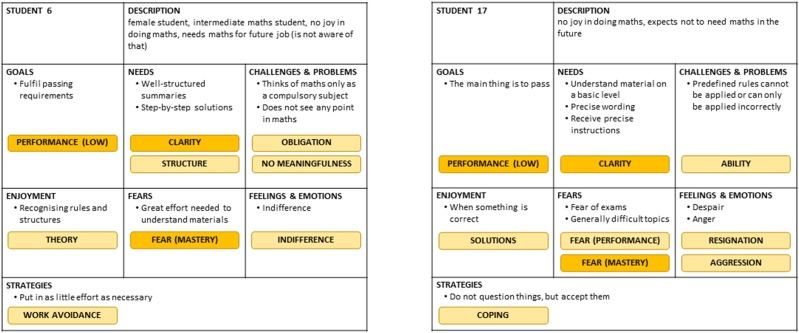
Example of tables which were assigned to the same cluster based on similar codes.

**Fig. 5:**
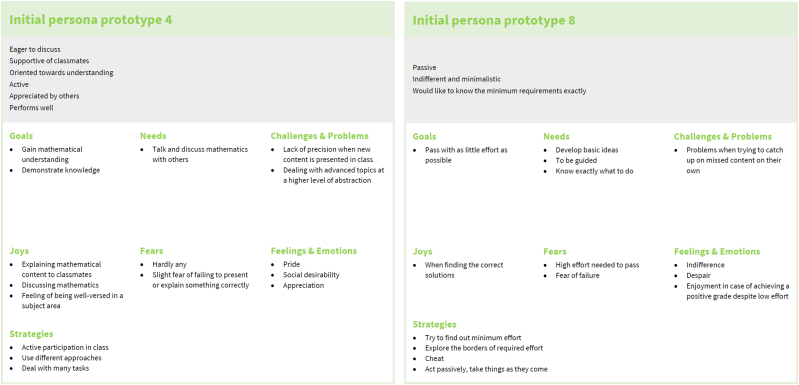
Examples of initial persona prototypes; all 21 initial persona prototypes are available as Multimedia component 2.

In the first step of the data processing, codes were created inductively. Examples for the resulting codes are as follows: goals: mastery, performance (high), performance (low); needs: materials, structure, support; challenges & problems: abstract nature of mathematics, transfer of theory into practice; joys: grades/feedback, solutions; fears: fear (performance), fear (mastery); feelings & emotions: indifference, resignation; strategies: work avoidance, cheating. The tables were clustered by similarities in the codes (see example in Fig. 4), for persona development. Then, a keyword and a brief description were assigned to each of the clusters. The resulting clusters formed initial prototypes of the personas (see examples in Fig. 5) and are attached to this work as Multimedia component 2.

**Fig. 6:**
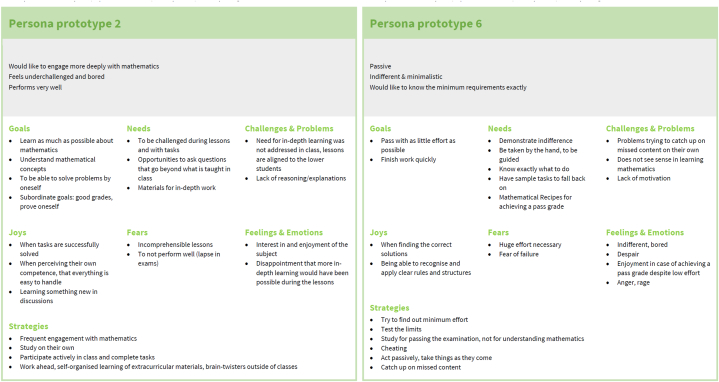
Examples of persona prototypes; all eleven persona prototypes are available as Multimedia component 3.

Persona prototypes were created individually by highlighting the similarities of each of the seven persona categories, thus increasing the level of abstraction of the personas. In this step, the initial prototypes were condensed to eleven persona prototypes (see examples in Fig. 6), which are attached to this work as Multimedia component 3.

**Fig. 7:**
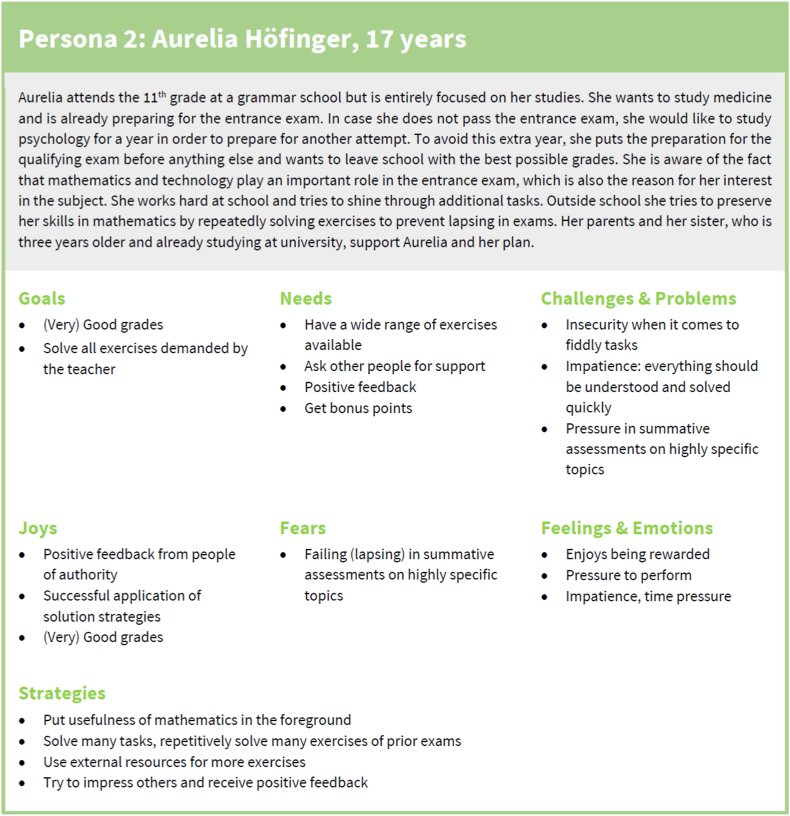
Example of a persona before validation; all five personas before validation are available as Multimedia component 4.

After the joint work of all researchers (see steps 3 and 4 in the methodology) new more extensive verbal descriptions of the personas were added based on the available information regarding persona categories, strategies, and the original verbal description. One example of the five personas developed in steps 3 and 4 is shown in Fig. 7; all five personas are attached as Multimedia component 4. As can be seen in Fig. 7, each persona contains a brief description of the background including age, career plans, wishes, and interest in mathematics in and outside school as well as their characteristics corresponding to the themes in Table 1. These personas were then subjected to a validation and improvement process.

The validation of these personas was carried out with 14 teachers and 83 students who had not been involved in the initial development of the personas. All teachers (100 %) and 79 students (95 %) reported that they recognised, if any at all, only minor flaws in the personas (e.g., several students added underachievement in exams as a fear of Aurelia Höfinger; one student added as a goal of Diana Markovic to “do well in written exams to avoid great effort for oral exams”). Agreement in the description of the personas was at least 90 %; in particular, they did not find important information missing in the description of the personas or aspects of the descriptions to be dispensable or irritating. Following Minichiello et al. [16], this indicates that the validity of the personas is satisfactory.

The good fit of the personas to the student population was also noted in the project FLINK and in the feedback of secondary school students. On the one hand, while collecting the written feedback, in 26 of the 83 feedbacks, no changes or additions were suggested; in 31 of the feedbacks, only minor additions or changes to the personas were suggested; four students reported that one specific persona—namely, Johannes Friedrich, an enquiring and keen mathematics student—did not seem like a real student. On the other hand, when collecting written feedback from secondary school students, there were intermittent exclamations such as “I know that one” or “That’s [name of a fellow student]!”. Such remarks were included in our field notes. Similarly, a good fit of the personas with the actual student population was reported in the interviews with in-service mathematics teachers.

### 4.2 Persona descriptions

*Johannes Friedrich.* Johannes is a student highly interested in mathematics who wants to gain as much mathematical knowledge as possible and is also interested in mathematical problems or concepts not presented in school. Johannes would like to discuss advanced mathematical problems or concepts with other students but rarely finds fellow students who share his interests. Johannes is often annoyed by the fact that standard problems are only solved for the next school test or the school-leaving examination and that there is no time for more in-depth study. His mathematical knowledge is often admired by his classmates, which, on the one hand, gives pleasure to him. On the other hand, this admiration also creates a certain amount of fear because, for Johannes, there would be few things worse than failing at a mathematical problem on the blackboard in the classroom.

*Aurelia Höfinger.* Aurelia is a student who usually gets very good or good grades on tests and exams in mathematics. To perform well, Aurelia invests a lot of time and tries to solve as many exercises as possible. In class, Aurelia works energetically, especially when her activities are rewarded with bonus points. Aurelia wants to learn new content as quickly and effectively as possible, regardless of the mathematical concepts. Problems that require the use of more complex mathematical concepts unsettle Aurelia. The biggest fear Aurelia has regarding mathematics lessons is that she will have a mental block in examinations or not be able to solve the problem and therefore receive grades other than A's.

*Manuel Winkler.* Manuel is a student with quite some potential in mathematics. However, the goals he sets for himself are so low that he can achieve them with little time and effort. For this reason, Manuel would like to have a mathematics lesson in which he is told exactly how many problems he has to solve and which method he has to use in order to receive a particular grade. To increase his effectiveness in studying, Manuel tries to develop a set of procedures for each mathematical concept that he can use to solve the problems. If Manuel cannot produce a recipe for a mathematical concept, he does not deal with this mathematical concept any further and tries to cheat in exams. Manuel invests more time and resources shortly before tests. However, he notices that having to fill gaps in his mathematical knowledge is becoming more challenging.

*Diana Markovic.* Diana invests a lot of time into learning mathematical concepts. However, she usually achieves poor grades on tests and finishes the school year with the lowest possible passing grade. Diana is aware of her weaknesses in mathematics and therefore tries to behave inconspicuously in mathematics lessons to avoid being called on by the teacher. Also, she rarely asks her classmates for help for fear of embarrassing herself. For studying, Diana mainly relies on the resources provided by her teacher. She tries to extract a set of procedures from these resources. Diana then uses these recipes to solve mathematics problems in class or on tests, even if they lead to meaningless results.

*Dominik Ghali.* Dominik knows that he should invest more effort into studying mathematics, hoping to take up a technical profession later on. At the moment, however, leisure activities are much more important to Dominik than doing well at school. This also applies to learning mathematics. Dominik wants to achieve good or average grades in mathematics, spending as little time studying as possible. This leads Dominik to focus on the most straightforward mathematical problems in class or exams. When Dominik is challenged by a mathematical problem, he quickly asks for help to solve that particular problem. For this reason, Dominik enjoys it less and less when a new mathematical concept is introduced or when he is asked to solve a mathematical problem in front of his classmates. While a year ago Dominik was quite calm before mathematics exams, they make him more and more anxious and his fear of failing is growing.

Each of these personas is representative of one of the five groups that we identified in our study. The names are chosen in a way suggestive of the background with which they are—typically or stereotypically—associated.

**Fig. 8:**
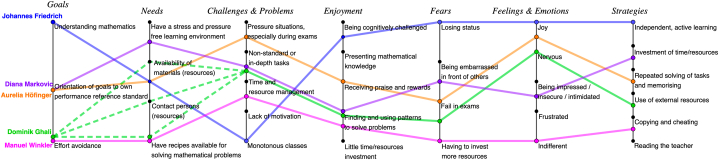
Overview of the assignment of the persona to the themes of each persona category.

In the following section, we describe themes (e.g., understanding mathematics) arising in each dimension of the personas (e.g., goals). We developed these themes by clustering similar bullet points in each dimension in the raw data and assigning a new keyword to each cluster. By clustering the bullet points, a higher level of abstraction could be achieved than is given by the dashboard personas. An overview of the resulting themes in each dimension is attached to this work as Multimedia component 6. The personas may be visualised and compared to one another with the help of these themes (see Fig. 8). We consider the main themes of each dimension as nominal. Even though they are not ranked, we present related themes in close proximity in the figure. This allows us to connect a persona to more than one theme within a dimension if needed. If a persona is related to two themes in the same dimension, we display these themes adjacent to each other in the figure and place the data point of the persona in the middle (see for example Diana concerning her needs). The only exception here is the assignment of Dominik Ghali in the dimension *Needs*, where three main themes have emerged. This is why the visualisation of Dominik Ghali’s persona is dashed in this area.

The coloured dots represent the allocation of the personas to the themes based on their characteristics. Each of the coloured lines in Fig. 8 represents one persona. Furthermore, the representation of the personas based on the themes of the individual categories (i.e., *Goals*, *Needs*, …) can be used, on the one hand, to validate the personas. This representation of the personas shows that the individual personas can be clearly distinguished from each other, although similarities occur at times. These differences between the personas support the assumption that the personas developed in our study represent distinct groups of mathematics students. On the other hand, further content-related insights can be drawn from the representation of the personas based on the themes of the individual categories. Thus, it can be seen that in some categories a kind of predominant theme can be established. We call those themes predominant which are central to at least three personas. For *Needs*, this is *Availability of materials (resources)*, for *Challenges & Problems* it is *Non-standard or in-depth tasks* and *Time and resource management*, for *Enjoyment* it is *Finding and using patterns to solve problems*, and for *Fears* it is *Failing in exams*.

In the following section, we describe how these personas differ from existing personas and how they can be used to support the development of technology-rich teaching and learning environments and resources.

## 5 Discussion

The goal of this study was to identify characteristics as well as prevalent combinations of such characteristics of Austrian academic upper secondary school mathematics students in their technology-rich environments. From the collection, analysis, and validation of administrative and secondary data on these students, we identified five prevalent combinations of student characteristics. Each of these five combinations of characteristics is presented by a persona, i.e., an archetypical and realistic representative of the cluster.

Research on aptitude-treatment interactions has not yet confirmed definite connections between student aptitudes and optimal teaching methods [[23], [24], [25],^55^]. Hunt [26] attributes a lack of results in this respect to an exaggerated focus on quantitative methods and concludes that statistical approaches are not sufficient to describe the relationship between student aptitudes and teaching methods. Thus, in our approach, we did not attempt to develop guidelines on how to teach students with specific aptitudes, but instead focused on various motivational and emotional aspects of students with regard to mathematics, i.e., the categories of the personas. We used a qualitative approach and aimed to systematically identify typical motivational and emotional characteristics of students and prevalent combinations thereof, and to present them in the form of personas to designers of technology and to teachers. These personas call upon the engineering expertise of designers and the pedagogical expertise of teachers, helping designers to create and helping teachers to choose learning resources that students can use to study according to their individual needs.

In this approach, we considered constructs such as goals, needs, challenges, or feelings associated with teaching and learning mathematics. Compared to works on aptitude-treatment interactions, our study is not based on manifest indicators. Instead, we used constructs to capture well-being and strategies of students related to learning mathematics. A major novelty in our study is that we present our results as personas rather than by statistical indicators.

International organisations stress the importance of the use of technologies in education [4]. To develop beneficial learning environments that implement these regulations and recommendations, it is necessary to have a sound knowledge of the characteristics and demands of students. Personas are an effective way to relate insights on these characteristics and demands to the practices of educators and of designers of educational technologies. The personas created in our study can be helpful for the development of technology-rich mathematics learning environments: On a small scale, for example, individual schools or teachers can make use of the personas when deciding on which technology to use for teaching and learning mathematics. On a large scale, the personas could, for example, inform the design of learning management systems or mathematical software. Compared to other UX methods such as usability tests or heuristic analyses [56], we chose personas as they are able to inform the design of technologies before or while they are being developed. By contrast, other methods are useful for evaluating technologies that have already been developed. We expect that consideration of the characteristics and needs of users is more readily implemented when creating prototypes rather than adapting essentially complete products, also due to the expenditure of resources.

In our study, we adapted techniques from UX research to develop personas of academic upper secondary school mathematics students in Austria. Future research could extend the evaluation of the effectiveness of personas in the field of education following the approach of Salminen et al. [14] by accompanying design processes in which the personas are used, such as the development of digital tools by technology developers or lesson design by teachers. Additionally, to address the lack of research on how well digital technologies are tailored to the needs of the learners (see, e.g., Ref. [35]), it would be valuable to assess usability (operability of the technical functions) and adequacy (appropriateness of content) of technologies developed using personas.

## 6 Conclusions, limitations, and future research

In this research, persona development techniques were applied in order to comprehensively capture the complex characteristics of mathematics students in upper secondary schools in Austria. Based on the results of our study, five distinct personas were identified which provide valuable insights into student goals, needs, challenges and problems, joys, fears, feelings and emotions, and strategies.

The strength of this work is its ability to identify and describe different types of academic upper secondary school mathematics students in Austria. The results may help teachers learn about the needs of their students and develop teaching strategies that address them, leading to better learning outcomes. Overall, the results of our research could be important as they highlight the different types of academic upper secondary mathematics students in Austria and provide valuable insights into their motivational profiles. We expect that the personas developed in our project can, on the one hand, support the development of user-centred educational technologies and, on the other hand, can be boundary objects to talk about issues in mathematics education and therefore serve, for example, learning settings in mathematics teacher education. Specifically, we consider the personas as a promising interactive tool to acquire knowledge related to the interplay of technology, pedagogy, and content as conceptualised by the TPACK model (see section 2.2).

Limitations of our results have to be considered regarding the geographical focus and the curricular requirements. It must be noted that the personas developed in our study are primarily applicable to school districts and to school types where the use of technologies is mandated by curricula in Austria. We consider this relevant since some of the profiles found in the target group in this project might not be prevalent in other school types due to students’ self-selection into upper secondary schools with or without a STEM focus.

Austria is a highly developed country and the socio-economic background of students in academic upper secondary schools is high compared to most other countries; their parents often have a higher level of education or professional training and a higher socio-economic status [50]. However, there are notable differences between different types of such schools, e.g., private schools or schools that attract mostly students from non-academic lower secondary schools (*Mittelschulen*). A possible selection bias that might occur due to the limited number of schools reached in our data collection can be moderated in future research by using alternative approaches in collecting data that facilitate including more schools. The findings can be generalised to student populations that have a similar background; future research can reveal if similar student profiles can be found in other student populations. To address the potential subjectivity in persona development, we carried out a comprehensive validation process which we expect to have reduced subjectivity in the personas.

In future studies, additional age groups should be considered. While our study focused exclusively on upper secondary school students, i.e., students in grades 9 to 12, expanding the student population to lower secondary school students, i.e., students in grades 5 to 8, could yield interesting results.

The data used for developing the personas in our study are based on secondary qualitative data obtained from a rather small-sized sample of 47 teachers and on administrative data. The inclusion of primary data, for instance, data collected from mathematics students themselves, as well as more extensive quantitative data should enrich the personas and further increase their validity. Furthermore, future studies should investigate to what extent the personas developed in our study could be related to existing characterisations of student groups regarding, for example, motivational aspects or mathematical competencies.

## Data availability

The data associated with this study has not been deposited into a publicly available repository. The datasets used and analysed during the current study are available from the corresponding author on reasonable request.

## Ethics statement

Participation in the study was voluntary and subject to informed consent.

## Funding

Open access funding provided by the 10.13039/501100003065University of Vienna.

## CRediT authorship contribution statement

**Robert Weinhandl:** Writing – original draft, Visualization, Methodology, Formal analysis, Data curation, Conceptualization. **Martin Mayerhofer:** Writing – review & editing, Formal analysis, Data curation. **Tony Houghton:** Writing – review & editing. **Zsolt Lavicza:** Writing – review & editing, Supervision, Methodology. **Lena Maria Kleinferchner:** Data curation. **Branko Anđić:** Writing – review & editing. **Michael Eichmair:** Writing – review & editing. **Markus Hohenwarter:** Supervision.

## Declaration of competing interest

The authors declare that they have no competing interests.

## Footnotes

1 “Lernen mit technologischer Unterstützung: Technologische Hilfsmittel sollen in allen Kompetenzbereichen sinnvoll zum Einsatz kommen. Sie müssen zumindest über grundlegende Funktionen zur Darstellung von Funktionen, Kurven und anderen geometrischen Objekten, zum symbolischen Umformen von Termen und Lösen von Gleichungen und Gleichungssystemen, zur Ermittlung von Ableitungs-und Stammfunktionen, zur Integration sowie zur Unterstützung bei Methoden und Verfahren in der Stochastik verfügen. Sachgerechtes und sinnvolles Nutzen technologischer Hilfsmittel durch geplantes Vorgehen ist sicherzustellen. Die minimale Realisierung besteht im Einsatz entsprechender Hilfsmittel beim Lösen von Aufgaben und dem gelegentlichen Einsatz als didaktisches Werkzeug beim Erarbeiten neuer Inhalte. Die maximale Realisierung ist der sinnvolle Einsatz derartiger Technologien als Werkzeug beim Modellieren, Visualisieren und Experimentieren.” [3].

2 “Die Minimalanforderungen an elektronische Hilfsmittel sind grundlegende Funktionen zur Darstellung von Funktionsgraphen, zum numerischen Lösen von Gleichungen und Gleichungssystemen, zur Ermittlung von Ableitungs-bzw. Stammfunktionen, zur numerischen Integration sowie zur Unterstützung bei Methoden und Verfahren in der Stochastik.“ [28].

3 Statistics Austria is the statistical office of the Republic of Austria.

4 Throughout the period between 2014 and 2019, the student population in Austrian academic upper secondary schools consisted of 37,400 to 39,700 students attending academic upper secondary schools (AHS-Oberstufe and Oberstufenrealgymnasien) in densely populated areas and of 40,900 to 42,400 students attending schools in intermediate density areas or thinly populated areas. The areas are categorised according to the urban-rural typology of Statistics Austria (https://www.statistik.at/web_en/classifications/regional_breakdown/urban_rural/index.html). The figures were extracted from databases of Statistics Austria using STATcube [52] and calculated using spreadsheets.
