# Supplementary material for: Enhancing user-centred educational design: Developing personas of mathematics school students
Source: Heliyon. 2024 Jan 7;10(2):e24173. doi: 10.1016/j.heliyon.2024.e24173 (PMC10827463; doi:10.1016/j.heliyon.2024.e24173)
Supplement: Multimedia component 1 [file mmc1.pdf]

Weinhandl, R., Mayerhofer, M., Houghton, T., Lavicza, Z., Kleinferchner, L. M., Anđić, B., Eichmair, M., Hohenwarter, M.

Enhancing user-centred educational design: Developing personas of mathematics school students

Multimedia component 1

Correspondence: Martin Mayerhofer, Department of Mathematics, Faculty of Mathematics, University of Vienna, Vienna, Austria, martin.mayerhofer@univie.ac.at

Student descriptions collected from in-service teachers

Student 1

Goals

- Highest possible understanding of the material

Needs

- Show links to previous knowledge or other areas of the subject matter
- Clear strategy to solve tasks of a certain type
- Comprehensible reasons for the choice of strategies
- Precise definitions and language

Challenges & Problems

- Solving unfamiliar problems
- Exercises requiring an experimental rather than a strategic approach

Joys

- Successfully applying memorised strategies to solve exercises

Fears

- No fears

Feelings & Emotions

- Joy

Strategies

- Ask specific questions in class when something is unclear
- Conscientious study of the material at home

Student 2

Goals

- Passing the entrance exam

Needs

- Individual support

Challenges & Problems

- In 10th and 11th grade, the curriculum prescribes a lot of content, so there is not always enough time to answer all questions.

Joys

- When working independently

Fears

- That due to distance learning the preparations for the school leaving examination are not ideal

Feelings & Emotions

- Joy when she has solved difficult tasks
- Demotivation, when many similar tasks have to be solved as an exercise.

Strategies

- Always asks many questions about tasks that might appear in a similar form in the entrance exam

Correspondence: Martin Mayerhofer, Department of Mathematics, Faculty of Mathematics, University of Vienna, Vienna, Austria, martin.mayerhofer@univie.ac.at

Student 3

Goals

- Extend knowledge
- Wants to present his knowledge

Needs

- Time to talk about maths

Challenges & Problems

- Grades do not always match the interest in the subject
- Sometimes too fast
- Lack of precision and accuracy

Joys

- Discussions about maths

Fears

- None

Feelings & Emotions

- Enjoying classes and being needed by classmates

Strategies

- High level of involvement in classroom activities

Student 4

Goals

- Writing a fairly good grade

Needs

- Do many exercises with a low level of complexity ("Typ-1-Aufgaben")
- Do sample tests

Challenges & Problems

- Very complex contexts cause difficulties

Joys

- When she has understood something

Fears

- Tasks with a higher level of complexity ("Typ-2-Aufgaben")

Feelings & Emotions

- Very neutral
- Happiness when she has understood something entirely

Strategies

- Study in a way to be able to pass an exam (rather than to comprehend the material)

Correspondence: Martin Mayerhofer, Department of Mathematics, Faculty of Mathematics, University of Vienna, Vienna, Austria, martin.mayerhofer@univie.ac.at

Student 5

Goals

- Understanding everything
- Getting to the bottom of everything

Needs

- Solve as many tasks as possible
- High work pace

Challenges & Problems

- No particular problems

Joys

- Presenting her solution to a problem in front of others

Fears

- Not getting an A on exams
- Failing in exams

Feelings & Emotions

- Enjoyment in classes
- Pressure to perform in exams

Strategies

- Tries to find a logical explanation for everything
- Tries to identify connections, links to other areas

Student 6

Goals

- Fulfil passing requirements

Needs

- Well-structured summaries
- Step-by-step solutions

Challenges & Problems

- Thinks of maths only as a compulsory subject
- Does not see any point in maths

Joys

- Recognising rules and structures

Fears

- Great effort needed to understand materials

Feelings & Emotions

- Indifference

Strategies

- Put in as little effort as necessary

Correspondence: Martin Mayerhofer, Department of Mathematics, Faculty of Mathematics, University of Vienna, Vienna, Austria, martin.mayerhofer@univie.ac.at

Student 7

Goals

- Good to average grade

Needs

- Lots of practice material
- Detailed, repeated explanations

Challenges & Problems

- Content is sometimes unclear
- Motivational problems with difficult topics

Joys

- Illustrative and interesting content that is clearly related to real life

Fears

- Not pass
- Afraid of not understanding new content

Feelings & Emotions

- Motivation when performing well and understanding material
- Frustration if content is not comprehensible at the first go

Strategies

- Raise the hand in class from time to time
- Study quite a lot before exams

Weinhandl, R., Mayerhofer, M., Houghton, T., Lavicza, Z., Kleinferchner, L. M., Anđić, B., Eichmair, M., Hohenwarter, M.
Enhancing user-centred educational design: Developing personas of mathematics school students: Multimedia component 1.
Correspondence: Martin Mayerhofer, Department of Mathematics, Faculty of Mathematics, University of Vienna, Vienna, Austria, martin.mayerhofer@univie.ac.at

Descriptions of former fellow students collected from pre-service teachers

Student 8

Goals

- Fulfil passing requirements

Needs

- Achieve the required minimum

Challenges & Problems

- Did not know how to do the tasks on his own
- Had problems how to approach exercises

Joys

- No joy at all

Fears

- That studying is useless
- Was very slow and therefore did not accomplish much

Feelings & Emotions

- Reassurance – repeatedly asks for basics in class and wants to be reassured that the recipe he has discovered actually works

Strategies

- Studies as much as possible
- Asks a lot of questions

Student 9

Goals

- Passing exams

Needs

- More material for visualisation (GeoGebra applets)
- Repeated explanations for connections between areas
- Receive help without having to ask for it

Challenges & Problems

- Could not see connections

Joys

- None

Fears

- Receive a failing grade

Feelings & Emotions

- Anxiety
- Mental block
- Discouraged

Strategies

- Memorise calculation patterns

Correspondence: Martin Mayerhofer, Department of Mathematics, Faculty of Mathematics, University of Vienna, Vienna, Austria, martin.mayerhofer@univie.ac.at

Student 10

Goals

- Make it through school

Needs

- Explanatory videos
- Group activities
- More time for questions
- More feedback
- Non-graded tests

Challenges & Problems

- Not able to follow the teacher's explanations
- Nature of the teacher's explanations does not correspond to her way of thinking
- Hardly any repetition in class
- No support from the teacher
- Does not ask questions as she cannot follow the explanations
- No guidance when studying
- Lack of sources of information

Joys

- When she can solve tasks herself

Fears

- Fear of failure

Feelings & Emotions

- Mainly negative
- Resignation
- Anger
- Rejection

Strategies

- Receives private tuition

Student 11

Goals

- During the lesson she did not pay attention and then complained that she did not understand anything

Needs

- Teacher authority

Challenges & Problems

- Learning everything on her own was almost impossible

Joys

- Talking with classmates

Fears

- None

Feelings & Emotions

- Despair

Strategies

- Study on her own when she did not understand material in class

Correspondence: Martin Mayerhofer, Department of Mathematics, Faculty of Mathematics, University of Vienna, Vienna, Austria, martin.mayerhofer@univie.ac.at

Student 12

Goals

- Successfully complete homework
- Pass exams

Needs

- Would like to ask questions about the subject matter, teacher is too impatient and does not answer questions as students should already know how to solve the respective problems
- Needs someone to call on and to ask comprehension questions without concerns

Challenges & Problems

- Homework is not discussed
- Exercise sheets are handed out as preparatory material before exams; students might be examined on anything covered by these exercise sheets
- No predefined goals

Joys

- Success in learning

Fears

- Teacher
- Failing grades

Feelings & Emotions

- Intimidated
- Frustrated
- Happy when being successful
- Interested and fast in comprehends material quickly in private tutoring

Strategies

- Receives private tuition

Student 13

Goals

- Passing grades

Needs

- Many explanations did not make sense

Challenges & Problems

- Lack of success results in a lack of motivation to study
- Easily distracted, not focused

Joys

- Not known

Fears

- Fear of failure

Feelings & Emotions

- Frustration
- Boredom
- Anger

Strategies

- Receives private tuition

Student 14

Goals

- Just pass
- Pass the school leaving examination

Needs

- Appealing and activating lessons (during teacher monologues she was always on her mobile phone)
- In the breaks before tests, she asked others for explanations and showed interest

Challenges & Problems

- In class she didn't have to pay attention or make a big effort
- She let things slide until shortly before exams and then could not catch up

Joys

- Conversations and exchange with classmates
- When things were explained to her, when she could ask questions and understood something little by little

Fears

- Fail to pass

Feelings & Emotions

- Interest
- Stress before tests

Strategies

- Cheats, "Study on the edge"

Student 15

Goals

- Fulfil passing requirements

Needs

- Counteract rigid memorisation already during the school year (fostered by adequate requirements in exams)

Challenges & Problems

- Basic knowledge was missing or was learnt out of context
- Knowledge not linked to other areas

Joys

- When there was a fixed calculation scheme

Fears

- Fear of the teacher
- That she could not understand the steps of calculation

Feelings & Emotions

- Fear
- Tension
- Nervousness

Strategies

- Step-by-step instructions for each type of exercises – memorise them before exams
- Understanding the mathematical content was not the primary goal (turned out to be a mistake as she had to repeat the school leaving examination twice)

Weinhandl, R., Mayerhofer, M., Houghton, T., Lavicza, Z., Kleinferchner, L. M., Anđić, B., Eichmair, M., Hohenwarter, M.
Enhancing user-centred educational design: Developing personas of mathematics school students: Multimedia component 1.
Correspondence: Martin Mayerhofer, Department of Mathematics, Faculty of Mathematics, University of Vienna, Vienna, Austria, martin.mayerhofer@univie.ac.at

Student 16

Goals

- Fulfil passing requirements

Needs

- Unknown

Challenges & Problems

- Comprehension reached limits in higher mathematics – linking knowledge across areas was difficult

Joys

- Not known

Fears

- Failing grade

Feelings & Emotions

- Relieved when receiving a pass grade and when an exam was over

Strategies

- Study for exams
- Seek help from classmates if needed

Student 17

Goals

- The main thing is to pass

Needs

- Understand material on a basic level
- Precise wording
- Receive precise instructions

Challenges & Problems

- Predefined rules cannot be applied or can only be applied incorrectly

Joys

- When something is correct

Fears

- Fear of exams
- Generally difficult topics

Feelings & Emotions

- Despair
- Anger

Strategies

- Do not question things, but accept them

Correspondence: Martin Mayerhofer, Department of Mathematics, Faculty of Mathematics, University of Vienna, Vienna, Austria, martin.mayerhofer@univie.ac.at

Student 18

Goals

- Fulfil passing requirements
- Not make a fool of themselves

Needs

- Explain content in detail and slowly enough so that there is no need to ask again
- Divide the class into groups and provide explanations to an individually tailored extent

Challenges & Problems

- Ashamed to ask questions
- Afraid of seeming stupid
- Some terms and ideas were not illustrative and understandable

Joys

- When studying together without being judged by anyone
- In tutoring with her classmates

Fears

- Look like a fool
- Fear of failure

Feelings & Emotions

- Fear
- Boredom
- Irritation
- Helplessness

Strategies

- Ask fellow students for private tutoring
- Put a lot of effort into preparation for exams

Student 19

Goals

- Advance to the next grade
- Become better in order to get a pass grade

Needs

- "Fear" of asking questions in class

Challenges & Problems

- Is doing quite well in other subjects, but difficulties in maths hinder advancing to the next grade

Joys

- Not known

Fears

- Fear of giving wrong answers
- Bad grades

Feelings & Emotions

- Fear of exams
- High respect for the subject – "all the maths is too difficult"

Strategies

- Seeks support from tutoring institute
- Learn a lot by himself/herself

Correspondence: Martin Mayerhofer, Department of Mathematics, Faculty of Mathematics, University of Vienna, Vienna, Austria, martin.mayerhofer@univie.ac.at

Student 20

Goals

- Just achieve a pass grade
- Advance to the next grade
- Pass the school leaving examination

Needs

- Advancement in class is too fast for her
- More time to practice
- Different exercises would be helpful when done together

Challenges & Problems

- Loss of joy for maths
- Exam stress becomes more and more

Joys

- None

Fears

- Repeatedly not pass
- Not be able to solve complex tasks or reverse calculation tasks

Feelings & Emotions

- Despair
- Anger

Strategies

- Try very hard
- Study for hours and hours

Student 21

Goals

- The main thing is to pass

Needs

- Didn't want help from classmates because she was too proud to admit that she was lagging behind and that someone else would do better than her

Challenges & Problems

- "Learning by heart" instead of learning to understand
- Goals set too low
- Looked for faults in the material and the teacher
- "I understand everything anyway, I don't know why it's not enough"

Joys

- Not known

Fears

- That she gets more and more confused

Feelings & Emotions

- Frustration
- Fear
- Disappointment

Strategies

- Practiced a lot
- Private tuition
- Memorising instead of trying to making sense of formulas or algorithms

Correspondence: Martin Mayerhofer, Department of Mathematics, Faculty of Mathematics, University of Vienna, Vienna, Austria, martin.mayerhofer@univie.ac.at

Student 22

Goals
- Pass grade

Needs
- None

Challenges & Problems
- Mathematics is not comprehensible
- Often did not find a suitable approach to solve problems

Joys
- Pass grade on exams

Fears
- Failing grade
- Having to repeat a form

Feelings & Emotions
- Stress
- Frustration
- Disinterest

Strategies
- Studying for many hours
- Private tuition

Student 23

Goals
- Achieve a pass grade
- School leaving examination
- Get through maths

Needs
- Wanted to know what maths would benefit him
- Tried to develop interest
- Explanation by classmates after professor’s explanations
- Could not see the connection between GeoGebra tasks and the maths learnt

Challenges & Problems
- He did not have a good idea about maths
- He understood only details and could not embed this in an overall knowledge

Joys
- When he achieved a pass grade
- When he understood something without help

Fears
- That he would be asked to repeat the content of the last lesson
- Before exams

Feelings & Emotions
- Pure despair
- Disillusionment when he understood something but then didn't work in a different context

Strategies
- Studied with classmates
- Could not follow mathematics professor

Correspondence: Martin Mayerhofer, Department of Mathematics, Faculty of Mathematics, University of Vienna, Vienna, Austria, martin.mayerhofer@univie.ac.at

Student 24

Goals
- To pass

Needs
- None

Challenges & Problems
- Internalising solution algorithms and learning by heart turned out to not lead to success in solving many tasks

Joys
- None

Fears
- Failure
- Not passing exams

Feelings & Emotions
- Frustration
- Insecurity

Strategies
- Memorise and hope

Student 25

Goals
- Pass grade

Needs
- Good and understandable explanations

Challenges & Problems
- The higher the grade the more the lack of mathematical knowledge increased

Joys
- None

Fears
- To not understand
- Failing grade

Feelings & Emotions
- Fear
- Annoyance
- Anger

Strategies
- Put moderate effort into studying

Correspondence: Martin Mayerhofer, Department of Mathematics, Faculty of Mathematics, University of Vienna, Vienna, Austria, martin.mayerhofer@univie.ac.at

Self-descriptions collected from pre-service teachers

Student 26

Goals

- To learn as much as possible about mathematics

Needs

- Lots of practice material

Challenges & Problems

- None – started at university to actually study materials

Joys

- There is always a clear result and you don't have to meet the teacher's taste

Fears

- Not living up to the reputation of always achieving an A

Feelings & Emotions

- Disappointment - we could have learned much more

Strategies

- Practise if necessary and do work in advance

Student 27

Goals

- Understand the system and the structure underlying the solution algorithms

Needs

- More difficult tasks
- Higher level of requirements in assessments

Challenges & Problems

- Language challenges (grew up trilingual)

Joys

- In creating/understanding new connections

Fears

- None – mistakes are part of the process of success

Feelings & Emotions

- Ambition
- Joy
- Sometimes frustration

Strategies

- Engage with the topic and link knowledge

Correspondence: Martin Mayerhofer, Department of Mathematics, Faculty of Mathematics, University of Vienna, Vienna, Austria, martin.mayerhofer@univie.ac.at

Student 28

Goals

- Solve problems in my own way

Needs

- Calculate mathematically *beautiful* tasks
- If I have questions they should be answered

Challenges & Problems

- None

Joys

- Exploring connections
- Developing solution strategies myself

Fears

- None

Feelings & Emotions

- Enjoyment
- Melancholy after this time

Strategies

- First try out on a note paper

Student 29

Goals

- To not lose the thread during class and to understand the tasks and topics

Needs

- I often asked questions when I wanted to know something more precisely or had not understood something
- I understood the material quite quickly and could have worked on it in greater detail - there was nothing offered

Challenges & Problems

- The teacher had difficulties with explaining some material; some students then asked me, which was why I couldn't hear what the teacher was saying

Joys

- When I had a correct result and had previously learnt the material without any help
- When I was able to help others understand and solve the tasks

Fears

- Losing the thread and not being able to follow the content anymore

Feelings & Emotions

- Mainly positive
- Also negative, because I was missing support and encouragement of the teacher most of the time

Strategies

- I tried to follow the lessons as well as possible
- Do my homework properly
- Ask other students if I had not understood something

Correspondence: Martin Mayerhofer, Department of Mathematics, Faculty of Mathematics, University of Vienna, Vienna, Austria, martin.mayerhofer@univie.ac.at

Student 30

Goals

- To understand the current material

Needs

- We were given guiding questions about the subject matter - I worked these out and studied them over and over again

Challenges & Problems

- Understanding complex issues
- Group work was very rare and I used to feel uncomfortable in it

Joys

- Explaining the content to classmates - understanding content better and consolidating the knowledge

Fears

- To present something on the blackboard that might not have been correct

Feelings & Emotions

- In lower secondary school, the whole situation at school was overwhelming
- In upper secondary school, my strengths came out
- Appreciation for helping others

Strategies

- Reviewing the material right before the lesson

Student 31

Goals

- To meet the requirements

Needs

- Good lessons were enough for me

Challenges & Problems

- None

Joys

- None

Fears

- None

Feelings & Emotions

- Rather positive

Strategies

- As little effort as possible

Correspondence: Martin Mayerhofer, Department of Mathematics, Faculty of Mathematics, University of Vienna, Vienna, Austria, martin.mayerhofer@univie.ac.at

Student 32

Goals

- To understand

Needs

- Sometimes explicit explanations needed
- Always learnt alone

Challenges & Problems

- Frustration when not able to understand

Joys

- When solving problems

Fears

- Of not understanding something

Feelings & Emotions

- Frustration at the beginning (of upper secondary school)
- Afterwards positive feelings

Strategies

- Being attentive

Student 33

Goals

- Good grades
- To understand everything

Needs

- Many exercises in all levels of difficulty
- Solving the tasks independently

Challenges & Problems

- I never really had any problems while learning; preparations consisted of explaining the material to classmates (which was the actual challenge for me)

Joys

- When there was a clear result in tasks and I arrived at the correct one
- Problem-solving tasks
- Learning new definitions and formulating them by myself

Fears

- There was none

Feelings & Emotions

- Very positive feelings
- Everything was simple and logical

Strategies

- Learning with the book
- Explanatory videos
- Paying attention in class

Weinhandl, R., Mayerhofer, M., Houghton, T., Lavicza, Z., Kleinferchner, L. M., Anđić, B., Eichmair, M., Hohenwarter, M.
Enhancing user-centred educational design: Developing personas of mathematics school students: Multimedia component 1.
Correspondence: Martin Mayerhofer, Department of Mathematics, Faculty of Mathematics, University of Vienna, Vienna, Austria, martin.mayerhofer@univie.ac.at

Student 34

Goals

- Good grades
- Pass the school leaving examination

Needs

- More attractive lessons
- Maths classes were pure monologues - couldn't pay attention for more than 10 minutes even though I was interested in maths

Challenges & Problems

- No relationships between the topics and no concepts were presented in the lessons
- Impression that maths is pure theory that can be learnt and that is useful for applications
- Only when learning, or 1 year later when new content was learnt, I saw the "big picture" and that everything is a logical construct and not as confusing as the individual topics were during the lessons

Joys

- When I studied at home, I understood the material and thought: this is not as complicated as the teacher explains and presents it

Fears

- That the tasks in exams are formulated in the exact same way as the explanations in class – incomprehensible

Feelings & Emotions

- Confusion and frustration – the lessons were not engaging
- Positive – studying at home was very informative and much more beneficial

Strategies

- Just study

Student 35

Goals

- To achieve good grades
- To understand contents

Needs

- I hardly had to study to understand the requirements
- Visualisation was important to me, to have all the content visually appealing

Challenges & Problems

- None

Joys

- When I could solve tasks in advance without specific mathematical knowledge
- Solving equations

Fears

- Of making unnecessary calculation mistakes in exams

Feelings & Emotions

- Enjoyment

Strategies

- Study without a strategy
- Get things done quickly

Correspondence: Martin Mayerhofer, Department of Mathematics, Faculty of Mathematics, University of Vienna, Vienna, Austria, martin.mayerhofer@univie.ac.at

Student 36

Goals
- Pass all exams
- School leaving examination

Needs
- More graphical representations/use of computers would have been helpful
- Many different exercises

Challenges & Problems
- Imagination quickly reached its limits
- Benefits were not always obvious

Joys
- That mathematics works according to "clear" rules, even if the path was not always unambiguous

Fears
- Not understanding the task correctly

Feelings & Emotions
- Positive, mathematics was generally easier to understand than other subjects

Strategies
- Learn skills that are necessary or helpful

Student 37

Goals
- Acquire a lot of knowledge
- Find many ways of solving a problem
- Linking knowledge

Needs
- Learning new things
- Multiple ways of solving problems
- Justify why something is the way it is
- Talking about mathematics

Challenges & Problems
- Sometimes it was not clear why some rules were applicable

Joys
- Solving equations

Fears
- None

Feelings & Emotions
- A good, joyful feeling

Strategies
- Ask
- Reverse thinking
- Discussion

Correspondence: Martin Mayerhofer, Department of Mathematics, Faculty of Mathematics, University of Vienna, Vienna, Austria, martin.mayerhofer@univie.ac.at

Student 38

Goals

- Study a lot
- Show that I can do something

Needs

- I wanted to be challenged to not get bored
- Tasks should have varied in difficulty more often so that some didn't require much effort, but others would take a bit longer

Challenges & Problems

- Sometimes difficult things were not given enough attention, but easier content was covered in too much detail

Joys

- When I was able to solve tasks independently and correctly all by myself

Fears

- That I wouldn't know what to do during exams
- Lapse of memory

Feelings & Emotions

- Interest
- Fascination

Strategies

- Explain as much as possible to myself and to understand it, even outside classes
- Not lose interest in mathematics by seeing difficult tasks more as a puzzle than as an impossible task

Student 39

Goals

- Good grades

Needs

- More frequent use of technology

Challenges & Problems

- Uncertainty if solutions were correct as no solution sheets were provided

Joys

- Proving mathematical statements on my own

Fears

- None

Feelings & Emotions

- Enjoyment
- Fun
- Joy of discovery

Strategies

- Active participation in class
- Studying

Correspondence: Martin Mayerhofer, Department of Mathematics, Faculty of Mathematics, University of Vienna, Vienna, Austria, martin.mayerhofer@univie.ac.at

Student 40

Goals
- To achieve good grades

Needs
- Graphic illustrations
- Comprehensible deductions

Challenges & Problems
- I found it particularly difficult to learn something when there was a lack of practical relevance

Joys
- Working independently with practical tasks
- Problem-solving tasks

Fears
- None

Feelings & Emotions
- Extremely positive

Strategies
- To be well prepared for the exams

Student 41

Goals
- To be as good as possible

Needs
- Was better when I understood things

Challenges & Problems
- In complex tasks, effort alone was not always enough

Joys
- When we were allowed to use GeoGebra because then I could visualise everything better (10th grade)

Fears
- Sequences/series were not my favourite topic

Feelings & Emotions
- Fun – when I had an idea of the things

Strategies
- Technology support (GeoGebra) made it easier for me to understand
- Understanding things - by studying a lot
- Seeking help from classmates

Correspondence: Martin Mayerhofer, Department of Mathematics, Faculty of Mathematics, University of Vienna, Vienna, Austria, martin.mayerhofer@univie.ac.at

Student 42

Goals
- To improve performance
- Graduate with an A

Needs
- Solve comprehension problems as often as necessary and in different ways

Challenges & Problems
- Having time to concentrate on mathematics in addition to other subjects
- He can only concentrate for a few hours and has to do other homework as well

Joys
- Positive and motivating feedback from the teacher
- Personal feedback on every test

Fears
- If I didn't understand everything at the beginning of a topic, that I might not be able to cope with the material in the future

Feelings & Emotions
- Positive feelings – very motivated teacher
- Success in learning
- Sense of achievement

Strategies
- "Practice makes perfect"
- Calculate standardised exercises recommended for exam preparation
- Write down necessary theoretical information

Student 43

Goals
- Develop a sound understanding

Needs
- To not learn by heart, but to understand
- Several explanations
- Exercises
- competent teacher that responds to questions

Challenges & Problems
- Inconclusive reasoning
- Had problems how to approach exercises

Joys
- Explaining mathematics to someone
- Understanding a topic well

Fears
- Never being afraid while learning

Feelings & Emotions
- Very positive
- Pride
- Some challenges

Strategies
- Think through and solve many tasks
- Choose different approaches

Correspondence: Martin Mayerhofer, Department of Mathematics, Faculty of Mathematics, University of Vienna, Vienna, Austria, martin.mayerhofer@univie.ac.at

Student 44

Goals

- Prepare for exams

Needs

- Use practical applications or visual representations to tackle challenging problems

Challenges & Problems

- Basic understanding of percentages
- Internalising calculation of percentages
- Summation and product formula

Joys

- Solving a task correctly and independently
- Being able to successfully explain my solution to others

Fears

- No fear
- Fun when learning

Feelings & Emotions

- Positive emotions
- No aversion

Strategies

- Understanding tasks

Student 45

Goals

- To get A's

Needs

- To have a good overview
- To prepare generally valid solution strategies so that I had a recipe for every type of task

Challenges & Problems

- Confusing notes
- Ready-made handouts helped me best

Joys

- Brain-teasers, e.g., finding general solution formulas

Fears

- Calculations with many large numbers that require the use of a calculator or CAS (I always entered numbers wrongly)

Feelings & Emotions

- Lower secondary school: I was bad at maths because I wasn't good at arithmetic computations
- When variables were introduced I got better and better

Strategies

- Study as much as I can until I can handle all the material
- Do all the homework
- I found it quite easy

Correspondence: Martin Mayerhofer, Department of Mathematics, Faculty of Mathematics, University of Vienna, Vienna, Austria, martin.mayerhofer@univie.ac.at

Student 46

Goals
- I just wanted to understand mathematics

Needs
- I didn't have anyone to ask when I got stuck

Challenges & Problems
- Abstracting

Joys
- Sense of achievement

Fears
- No fear when learning

Feelings & Emotions
- Injustice

Strategies
- None

Student 47

Goals
- Good grades

Needs
- Good explanations
- Study materials

Challenges & Problems
- Mathematical terminology

Joys
- That there is always a clear result

Fears
- No fears

Feelings & Emotions
- Enjoyment

Strategies
- Study diligently
