# Supplementary material for: Enhancing user-centred educational design: Developing personas of mathematics school students
Source: Heliyon. 2024 Jan 7;10(2):e24173. doi: 10.1016/j.heliyon.2024.e24173 (PMC10827463; doi:10.1016/j.heliyon.2024.e24173)
Supplement: Multimedia component 2 [file mmc2.pdf]

Weinhandl, R., Mayerhofer, M., Houghton, T., Lavicza, Z., Kleinferchner, L. M., Anđić, B., Eichmair, M., Hohenwarter, M.

Enhancing user-centred educational design: Developing personas of mathematics school students

Multimedia component 2

Correspondence: Martin Mayerhofer, Department of Mathematics, Faculty of Mathematics, University of Vienna, Vienna, Austria, martin.mayerhofer@univie.ac.at

Initial persona prototype 1

Wants to learn a lot and wants good grades as confirmation

Uses tools independently to understand content better

Picks up new content quickly

Goals

- Understand content as well as possible
- Achieve as good grades as possible

Needs

- Illustration of content using technological aids
- wide range of exercises at different levels of difficulty

Challenges & Problems

- Imagination reaches its limits in more complex tasks
- Uncertainty due to more complex tasks

Joys

- When content becomes clear by switching to graphical representations

Fears

- None

Feelings & Emotions

- Enjoyment of the subject matter
- Mathematics feels logical

Strategies

- Consciously acquire learning strategies:
    - Frequent activity and repetition
    - View teaching as a resource for personal development
    - Use external resources (classmates, learning videos, technology)

Weinhandl, R., Mayerhofer, M., Houghton, T., Lavicza, Z., Kleinferchner, L. M., Anđić, B., Eichmair, M., Hohenwarter, M.
Enhancing user-centred educational design: Developing personas of mathematics school students: Multimedia component 2.
Correspondence: Martin Mayerhofer, Department of Mathematics, Faculty of Mathematics, University of Vienna, Vienna, Austria, martin.mayerhofer@univie.ac.at

Initial persona prototype 2

Wants to learn mathematics profoundly

Seeks opportunities that promote deeper understanding

Feels underchallenged and bored in mathematics classes

Goals

- Main objective: to learn as much as possible about mathematics
- Subordinate goals: good grades, prove their own ability

Needs

- Materials for in-depth learning
- Opportunities to ask questions that go beyond the material taught in school
- Be challenged

Challenges & Problems

- Need for in-depth learning was not addressed in class

Joys

- When tasks are successfully solved
- When perceiving their own competence

Fears

- Incomprehensible lessons
- Not achieve *very* good results

Feelings & Emotions

- Interest in the subject
- Enjoyment that everything is easy to handle
- Disappointment that more in-depth learning would be possible

Strategies

- Frequent occupation with mathematics
- Active participation in lessons
- Work ahead
- Independent study to deepen knowledge

Correspondence: Martin Mayerhofer, Department of Mathematics, Faculty of Mathematics, University of Vienna, Vienna, Austria, martin.mayerhofer@univie.ac.at

Initial persona prototype 3

Enjoys engaging in mathematics

Performs very well

Does not have to make a lot of effort to follow classes

Goals

- Acquire extensive knowledge specific to mathematics

Needs

- Recognise and enjoy the beauty of mathematics
- Recognise connections
- Be challenged

Challenges & Problems

- Hardly any problems at all
- Slight problems when strategies are missing

Joys

- Recognising mathematical connections
- Successfully applying solution strategies

Fears

- None

Feelings & Emotions

- Ambition
- Enjoyment

Strategies

- Use resources available at school: lessons, ask teacher
- Recognise and use own competence and confidence with regard to mathematics
- Work conscientiously and engage frequently in the subject

Correspondence: Martin Mayerhofer, Department of Mathematics, Faculty of Mathematics, University of Vienna, Vienna, Austria, martin.mayerhofer@univie.ac.at

Initial persona prototype 4

Eager to discuss
Supportive of classmates
Oriented towards understanding
Active
Appreciated by others
Performs well

Goals

- Gain mathematical understanding
- Demonstrate knowledge

Needs

- Talk and discuss mathematics with others

Challenges & Problems

- Lack of precision when new content is presented in class
- Dealing with advanced topics at a higher level of abstraction

Joys

- Explaining mathematical content to classmates
- Discussing mathematics
- Feeling of being well-versed in a subject area

Fears

- Hardly any
- Slight fear of failing to present or explain something correctly

Feelings & Emotions

- Pride
- Social desirability
- Appreciation

Strategies

- Active participation in class
- Use different approaches
- Deal with many tasks

Correspondence: Martin Mayerhofer, Department of Mathematics, Faculty of Mathematics, University of Vienna, Vienna, Austria, martin.mayerhofer@univie.ac.at

Initial persona prototype 5

Places high marks above comprehensive understanding

Wants to be noticed in a positive way

Performs well

Wants content to be presented in a ready-to-use manner

Goals

- Achieve good grades
- Do well in exams

Needs

- Solution strategies
- Automate ways of solving problems
- Visualisation
- Learning materials that provide a guideline (mathematical derivations, explanations, and recipes)

Challenges & Problems

- When ready-made materials are missing
- When materials are confusing
- When dealing with technical language and abstraction

Joys

- In successfully solving tasks
- Positive feedback

Fears

- Hardly any

Feelings & Emotions

- Enjoyment of a sense of achievement
- Mainly positive emotions

Strategies

- Learn until ways of solving problems are internalised (“Practice makes perfect”)
- Prepare well for exams
- Try to anticipate exam tasks

Correspondence: Martin Mayerhofer, Department of Mathematics, Faculty of Mathematics, University of Vienna, Vienna, Austria, martin.mayerhofer@univie.ac.at

Initial persona prototype 6

Rather low level of performance

Tension

Hopes that knowledge of mathematical recipes will be enough

Uses additional resources as a support

Goals

- Positive grades in exams and school reports

Needs

- Materials and technological tools for illustration and support

Challenges & Problems

- In transferring memorised solution strategies to new tasks
- Connections not comprehensible

Joys

- Hardly any
- When fixed mathematical recipes lead to the solution

Fears

- Failing grades
- Fail in exams

Feelings & Emotions

- Nervousness
- Pressure, tension
- Fear
- Blockade
- Frustration

Strategies

- Learning calculation patterns, learning by heart
- Study for exams
- Ask classmates for help

Weinhandl, R., Mayerhofer, M., Houghton, T., Lavicza, Z., Kleinferchner, L. M., Anđić, B., Eichmair, M., Hohenwarter, M.

Correspondence: Martin Mayerhofer, Department of Mathematics, Faculty of Mathematics, University of Vienna, Vienna, Austria, martin.mayerhofer@univie.ac.at

Initial persona prototype 7

Student with average performance

Does everything she is told to do – no more, no less

Unemotional

Goals

- Get average grades
- Average achievement compared to others

Needs

- Exercises
- Lots of explanations
- May need to acquire basic skills

Challenges & Problems

- Complex topics cause technical and motivational problems

Joys

- When illustration leads to better understanding
- When there are links to everyday life

Fears

- Insufficient performance
- Complex tasks and contents

Feelings & Emotions

- Neutral

Strategies

- Try to anticipate exam tasks
- Make effort to a certain extent

Weinhandl, R., Mayerhofer, M., Houghton, T., Lavicza, Z., Kleinferchner, L. M., Anđić, B., Eichmair, M., Hohenwarter, M.

Correspondence: Martin Mayerhofer, Department of Mathematics, Faculty of Mathematics, University of Vienna, Vienna, Austria, martin.mayerhofer@univie.ac.at

Initial persona prototype 8

Passive

Indifferent and minimalistic

Would like to know the minimum requirements exactly

Goals

- Pass with as little effort as possible

Needs

- Develop basic ideas
- To be guided
- Know exactly what to do

Challenges & Problems

- Problems when trying to catch up on missed content on their own

Joys

- When finding the correct solutions

Fears

- High effort needed to pass
- Fear of failure

Feelings & Emotions

- Indifference
- Despair
- Enjoyment in case of achieving a positive grade despite low effort

Strategies

- Try to find out minimum effort
- Explore the borders of required effort
- Cheat
- Act passively, take things as they come

Weinhandl, R., Mayerhofer, M., Houghton, T., Lavicza, Z., Kleinferchner, L. M., Anđić, B., Eichmair, M., Hohenwarter, M.
Enhancing user-centred educational design: Developing personas of mathematics school students: Multimedia component 2.
Correspondence: Martin Mayerhofer, Department of Mathematics, Faculty of Mathematics, University of Vienna, Vienna, Austria, martin.mayerhofer@univie.ac.at

Initial persona prototype 9

Lags behind in class

Tries to find a less onerous workaround in order to avoid constant studying

Tries to force a positive grade with diligence, patience, and private tutoring before exams

Rarely gets grades better than the lowest pass grade

Goals

- Get pass grades

Needs

- Slow work pace
- Sufficient number of exercises
- Sufficiently extensive explanations

Challenges & Problems

- Frequently has problems how to approach exercises

Joys

- Hardly present
- When getting pass grades

Fears

- Failing grades
- Study without getting rewarded in exams
- Fear of failure

Feelings & Emotions

- Irritation
- Helplessness
- Despair
- Frustration

Strategies

- Trying to force a positive grade through extensive study and effort
- Private tutoring

Weinhandl, R., Mayerhofer, M., Houghton, T., Lavicza, Z., Kleinferchner, L. M., Anđić, B., Eichmair, M., Hohenwarter, M.
Enhancing user-centred educational design: Developing personas of mathematics school students: Multimedia component 2.
Correspondence: Martin Mayerhofer, Department of Mathematics, Faculty of Mathematics, University of Vienna, Vienna, Austria, martin.mayerhofer@univie.ac.at

Initial persona prototype 10

Student with weak to average performance who is afraid that his weaknesses will be discovered
Often female

Goals

- Do better in the next exam – achieve a pass grade
- Finish the year with a desired grade

Needs

- Detailed and slow explanations by the teacher
- A range of study materials for independent learning
- More time to study and to work on tasks

Challenges & Problems

- To get help when there were problems when studying or when trying to solve exercises
- Organising additional study materials
- Cannot find causes for mistakes or wrong results

Joys

- When learning or solving tasks can be done without being assessed
- When tasks can be solved without external help or asking others
- When studying together with a private tutor

Fears

- Failing in the next assessment or exam
- Having to ask the teacher or classmates for help

Feelings & Emotions

- Fear
- Respect – mathematics is important and difficult
- Being at the mercy of mathematics

Strategies

- Private tutoring
- Put a lot of time and effort into mathematics
- Memorise patterns

Weinhandl, R., Mayerhofer, M., Houghton, T., Lavicza, Z., Kleinferchner, L. M., Anđić, B., Eichmair, M., Hohenwarter, M.
Enhancing user-centred educational design: Developing personas of mathematics school students: Multimedia component 2.
Correspondence: Martin Mayerhofer, Department of Mathematics, Faculty of Mathematics, University of Vienna, Vienna, Austria, martin.mayerhofer@univie.ac.at

Initial persona prototype 11

Believes he will never need the subject or the content of mathematics again

Blocks, is actively against mathematics

Goals

- Only wants a pass grade
- Get over mathematics quickly

Needs

- Any tool that helps pass the next exam

Challenges & Problems

- No motivation for mathematics
- Low participation in class
- no sense of achievement

Joys

- Pass grade
- When studying for an exam is done

Fears

- Getting a failing grade
- That mathematics will be more difficult and time-consuming in the future

Feelings & Emotions

- Anger, rage
- Boredom because it is pointless

Strategies

- Study for exams, but not too much

Correspondence: Martin Mayerhofer, Department of Mathematics, Faculty of Mathematics, University of Vienna, Vienna, Austria, martin.mayerhofer@univie.ac.at

Initial persona prototype 12

Crams just before exams

Goals

- Achieve the desired grade
- Pass the next assessment or exam

Needs

- A variety of opportunities for studying
- That teachers or classmates are available to answer questions

Challenges & Problems

- Study and revise all the topics before the tests
- Make connections between topics
- Applications of mathematics in non-typical contexts

Joys

- Aha experiences
- When an exam is graded according to their expectations

Fears

- To not pass

Feelings & Emotions

- Stress especially before exams
- Relief when an exam is over – again relief when the grade is alright

Strategies

- Cheating and “muddling through”
- “Study on the edge”: Enormous study effort shortly before exams

Correspondence: Martin Mayerhofer, Department of Mathematics, Faculty of Mathematics, University of Vienna, Vienna, Austria, martin.mayerhofer@univie.ac.at

Initial persona prototype 13

Wants to have help and feedback readily available

Goals

- To be able to pass exams, assessments, homework and other assignments in an appropriate way

Needs

- Would like to be given time for more in-depth questions and explanations
- Would also like to know why something needs to be learnt and why it is important at some point

Challenges & Problems

- When the aim or the task is not clear
- Problems making intra-mathematical connections and connections between mathematics and everyday life

Joys

- Success in assessments, exams, and homework

Fears

- Not understanding everything, not getting the big picture
- Not being able to connect knowledge
- Fail in tests, assessments, homework and other assignments

Feelings & Emotions

- Awe of mathematics
- Disillusionment or even resentment when self-developed connections do not fit; when the own way of solving a task is not correct

Strategies

- Study a lot and make use of all study resources

Correspondence: Martin Mayerhofer, Department of Mathematics, Faculty of Mathematics, University of Vienna, Vienna, Austria, martin.mayerhofer@univie.ac.at

Initial persona prototype 14

Memorises mathematical recipes

Goals

- Pass the next exam and finish the school year with a pass grade

Needs

- Clear instructions on how to do things
- Clear step-by-step approach when solving tasks
- To have sample solutions and sample strategies for solving exercises

Challenges & Problems

- Memorises recipes but has troubles to adapt these recipes or make connections between recipes
- Connections are not identified and understood
- Problem with non-standard tasks

Joys

- When they recognise in an exercise that a known scheme can be used
- When a result is correct

Fears

- Fear of difficult and new topics
- Fear of applications outside the field of mathematics

Feelings & Emotions

- Fear
- Despair
- Tension
- Nervousness

Strategies

- Developing patterns and instructions for solving exercises
- Memorising patterns and instructions

Weinhandl, R., Mayerhofer, M., Houghton, T., Lavicza, Z., Kleinferchner, L. M., Anđić, B., Eichmair, M., Hohenwarter, M.
Enhancing user-centred educational design: Developing personas of mathematics school students: Multimedia component 2.
Correspondence: Martin Mayerhofer, Department of Mathematics, Faculty of Mathematics, University of Vienna, Vienna, Austria, martin.mayerhofer@univie.ac.at

Initial persona prototype 15

Finds it cool to be bad at maths

Goals

- Pass with as little effort as necessary

Needs

- Show that they are cool because they don't like or are not good at mathematics

Challenges & Problems

- Learning what you missed on your own

Joys

- Boasting with not being good at mathematics

Fears

- None

Feelings & Emotions

- Despair when having to catch up on missed content, when time was too short to be prepared for exams or when something could not be understood

Strategies

- Catch up on missed content

Correspondence: Martin Mayerhofer, Department of Mathematics, Faculty of Mathematics, University of Vienna, Vienna, Austria, martin.mayerhofer@univie.ac.at

Initial persona prototype 16

Performs well and wants to show this

Goals

- To develop an understanding of mathematics

Needs

- Different explanations
- Have their questions answered
- Be able to also ask in-depth questions and receive detailed explanations

Challenges & Problems

- Inconclusive solutions to a task or incomprehensible proofs

Joys

- Being able to talk about/discuss mathematics and being able to explain maths to a classmate

Fears

- None

Feelings & Emotions

- Positive
- Proud of achievements (grades or having explained something to a classmate)

Strategies

- Develop and use different approaches and strategies

Correspondence: Martin Mayerhofer, Department of Mathematics, Faculty of Mathematics, University of Vienna, Vienna, Austria, martin.mayerhofer@univie.ac.at

Initial persona prototype 17

Hard-working

Goals

- Get a pass grade
- Improve the grade compared to the grade in the prior assessment

Needs

- To get lots of practice time and lots of practice materials

Challenges & Problems

- In non-standard exercises or when tasks are extensive and knowledge and skills from different areas are needed

Joys

- Good grade
- When many tasks have been solved

Fears

- That the investment or input (time and tasks) will not be rewarded
- Fear of extensive and complex tasks

Feelings & Emotions

- Uncertainty: When have I spent sufficient time studying? When have I understood maths well enough?
- Enjoyment and pride when they receive a good grade or solve many exercises

Strategies

- Memorise solution strategies for each type of exercises
- Study for many hours, solve many exercises
- Private tutoring
- Do homework and solve additional exercises

Correspondence: Martin Mayerhofer, Department of Mathematics, Faculty of Mathematics, University of Vienna, Vienna, Austria, martin.mayerhofer@univie.ac.at

Initial persona prototype 18

Good student

Externally regulated: acts under orders

Goals

- To achieve A's, to get good marks
- To be able to understand everything and solve all (given) tasks

Needs

- To have someone to ask and discuss problems with
- Have lots of specific practice materials readily available
- Works on his/her own

Challenges & Problems

- The amount of subjects they want/need to perform well in (see goals)
- When other students interfere during lessons; being distracted from learning

Joys

- Achieving a good grade
- When they receive feedback that a task has been solved correctly
- When they can show their knowledge
- Praise/feedback from the teacher
- When they did not need any help to reach a goal

Fears

- To not understand something (especially when a topic is newly introduced)
- To not live up to their reputation and to other's expectations
- To not achieve an A or a good grade in an exam; to fail especially on summative performance assessments

Feelings & Emotions

- Impatience – wants things to be done and then to receive immediate feedback
- Highly enjoys having success
- Fear of not achieving a good grade
- Pressure to perform in summative assessments

Strategies

- Solve many tasks, also use other sources (standardised exercises or videos)
- Always participate and pay attention in class, do homework conscientiously
- Trying to do diligent work

Initial persona prototype 19

Performs well and is full of curiosity

Goals

- Learn a lot and widen knowledge
- Understand mathematics and mathematical concepts
- Be able to show what they can do
- Achieve good grades
- Be able to solve exercises in their own way

Needs

- To be challenged during lessons and with tasks.
- To be able to participate in class
- To talk about mathematics (with the teacher)
- Precise and purposeful language and teaching
- To get to know many ways of solving problems and applications

Challenges & Problems

- If lessons did not have a specific goal or practical relevance
- Underchallenged, classes only focused on easy tasks and underperforming students
- When the big picture and the relation of a topic to it is missing
- When reasons and explanations are missing

Joys

- Independent work
- Solving complex or practical problems
- Mathematical conversations and discussions
- Develop own strategies and solutions

Fears

- Lapse in exams or discussions

Feelings & Emotions

- Enjoyment of classes and mathematics
- Interested in and fascinated by mathematics

Strategies

- Independent study
- Engaging in brain-twisters also outside school
- Participating in lessons and completing tasks to the best of their ability

Weinhandl, R., Mayerhofer, M., Houghton, T., Lavicza, Z., Kleinferchner, L. M., Anđić, B., Eichmair, M., Hohenwarter, M.
Enhancing user-centred educational design: Developing personas of mathematics school students: Multimedia component 2.
Correspondence: Martin Mayerhofer, Department of Mathematics, Faculty of Mathematics, University of Vienna, Vienna, Austria, martin.mayerhofer@univie.ac.at

Initial persona prototype 20

Average performance but not involved in mathematics

Mathematics doesn't matter to them

Goals

- Achieve pass grades
- Pass with as little effort as necessary

Needs

- Sample exercises and solutions
- Well-structured summaries and explanations (reduced to essential aspects)

Challenges & Problems

- Maths problems are increasing → fundamentals are becoming more and more difficult to understand
- Meaningfulness of mathematics decreases in their eyes

Joys

- Being able to recognise and apply clear rules and structures

Fears

- Not being able to understand something or to solve the next problem
- That the required effort becomes more or too much

Feelings & Emotions

- Emotionless towards maths unless there is a particularly bad (or a particularly good) grade

Strategies

- Use time in the most efficient way: as little input (time/task/...) as necessary for sufficient output

Weinhandl, R., Mayerhofer, M., Houghton, T., Lavicza, Z., Kleinferchner, L. M., Anđić, B., Eichmair, M., Hohenwarter, M.
Enhancing user-centred educational design: Developing personas of mathematics school students: Multimedia component 2.
Correspondence: Martin Mayerhofer, Department of Mathematics, Faculty of Mathematics, University of Vienna, Vienna, Austria, martin.mayerhofer@univie.ac.at

Initial persona prototype 21

Sees mathematics as a means to an end

Goals

- Achieve the best possible grade
- Manage the next challenge (university entrance examination)
- Perform well in the school leaving examination, so that then they can apply for a good university/...

Needs

- Many different exercises
- Get clear guidelines and structures
- Develop mathematical recipes on their own
- Specific practice for exams

Challenges & Problems

- Actual benefit is not realised
- Cooperation with others, when he is not pursuing his own goal in group work
- When he does not get opportunities to express his concerns

Joys

- When (he thinks) he has understood something; when he has a strategy to adhere to
- When he can work alone on his own goal and achieve this goal

Fears

- To not understand content or a task correctly
- To be in the centre of attention or in front of the class
- Complex tasks that are not straight forward
- That preparation for exams (school or university) is not ideal due to external circumstances

Feelings & Emotions

- Good when he receives appreciation
- Enjoyment when he has solved or achieved something

Strategies

- Study what is necessary → focuses on the result of the exam
- Lots of repetition and practice
- Do homework/exercises and participate in class (to avoid having to put in effort later)
