# Supplementary material for: Enhancing user-centred educational design: Developing personas of mathematics school students
Source: Heliyon. 2024 Jan 7;10(2):e24173. doi: 10.1016/j.heliyon.2024.e24173 (PMC10827463; doi:10.1016/j.heliyon.2024.e24173)
Supplement: Multimedia component 3 [file mmc3.pdf]

Weinhandl, R., Mayerhofer, M., Houghton, T., Lavicza, Z., Kleinferchner, L. M., Anđić, B., Eichmair, M., Hohenwarter, M.

Enhancing user-centred educational design: Developing personas of mathematics school students

Multimedia component 3

Correspondence: Martin Mayerhofer, Department of Mathematics, Faculty of Mathematics, University of Vienna, Vienna, Austria, martin.mayerhofer@univie.ac.at

Persona prototype 1

High self expectations (knowledge gains & performance)

Picks up new content quickly

Goals

- Very good grades
- Acquire extensive specialised knowledge
- Be able to solve tasks

Needs

- To recognise the beauty of mathematics
- Talk to others about mathematical problems
- Wide range of exercises

Challenges & Problems

- Complex tasks sometimes lead to uncertainty

Joys

- Good grades and feedback
- Recognising mathematical connections
- Successful application of solution strategies

Fears

- Reputation could be lost
- Being able to perform well in every examination

Feelings & Emotions

- Enjoyment of mathematics
- Enjoyment of a sense of achievement
- Pressure to perform and time pressure: Everything should be done sufficiently and very well

Strategies

- Complete tasks conscientiously
- Frequent engagement and repetition
- Perceive teaching as a resource for personal development
- Use external resources (classmates, learning videos, technology)

Correspondence: Martin Mayerhofer, Department of Mathematics, Faculty of Mathematics, University of Vienna, Vienna, Austria, martin.mayerhofer@univie.ac.at

Persona prototype 2

Would like to engage more deeply with mathematics

Feels underchallenged and bored

Performs very well

Goals

- Learn as much as possible about mathematics
- Understand mathematical concepts
- To be able to solve problems by oneself
- Subordinate goals: good grades, prove oneself

Needs

- To be challenged during lessons and with tasks
- Opportunities to ask questions that go beyond what is taught in class
- Materials for in-depth work

Challenges & Problems

- Need for in-depth learning was not addressed in class, lessons are aligned to the lower students
- Lack of reasoning/explanations

Joys

- When tasks are successfully solved
- When perceiving their own competence, that everything is easy to handle
- Learning something new in discussions

Fears

- Incomprehensible lessons
- To not perform well (lapse in exams)

Feelings & Emotions

- Interest in and enjoyment of the subject
- Disappointment that more in-depth learning would have been possible during the lessons

Strategies

- Frequent engagement with mathematics
- Study on their own
- Participate actively in class and complete tasks
- Work ahead, self-organised learning of extracurricular materials, brain-twisters outside of classes

Weinhandl, R., Mayerhofer, M., Houghton, T., Lavicza, Z., Kleinferchner, L. M., Anđić, B., Eichmair, M., Hohenwarter, M.
Enhancing user-centred educational design: Developing personas of mathematics school students: Multimedia component 3.
Correspondence: Martin Mayerhofer, Department of Mathematics, Faculty of Mathematics, University of Vienna, Vienna, Austria, martin.mayerhofer@univie.ac.at

Persona prototype 3

Performs well and wants to show this

Supports classmates

Oriented towards understanding

Active

Appreciated by others

Performs well

Goals

- Gain professional understanding
- Present knowledge

Needs

- To talk about and discuss mathematics with others
- In-depth engagement with mathematics
- Receive different explanations

Challenges & Problems

- Lack of precision when teacher presents new content in class
- When dealing with advanced topics at a higher level of abstraction
- Inconclusive solutions

Joys

- Explaining maths to classmates
- Discussing mathematics
- Feeling of being well-versed in a mathematical topic

Fears

- Hardly any
- Slight fear of presenting or explaining something incorrectly

Feelings & Emotions

- Pride
- Social inclusion
- Appreciation

Strategies

- Active participation in class
- Use different approaches
- Deal with many tasks

Weinhandl, R., Mayerhofer, M., Houghton, T., Lavicza, Z., Kleinferchner, L. M., Anđić, B., Eichmair, M., Hohenwarter, M.
Enhancing user-centred educational design: Developing personas of mathematics school students: Multimedia component 3.
Correspondence: Martin Mayerhofer, Department of Mathematics, Faculty of Mathematics, University of Vienna, Vienna, Austria, martin.mayerhofer@univie.ac.at

Persona prototype 4

Places high marks above comprehensive understanding

Wants to be have a positive reputation

Wants content to be presented in a ready-to-use manner

Wants support on demand

Mathematics as a means to an end

Goals

- Achieve average or good grades
- Do well in exams
- Be able to do homework adequately

Needs

- Memorising solution strategies
- Visualisation
- Study materials that provide a guideline (mathematical derivations, explanations, recipes)
- Practice a lot

Challenges & Problems

- When ready-made materials are missing
- When materials are confusing
- When having to deal with technical language and abstraction
- When clear instructions are missing

Joys

- Successfully solving (parts of) exercises
- Positive feedback/grades

Fears

- Hardly any
- To not be able to make mathematical connections
- Failing in exams
- That the effort does not pay off

Feelings & Emotions

- Enjoyment of a sense of achievement
- Mainly positive emotions
- Uncertainty as to whether effort is enough

Strategies

- Study until solution strategies are internalised ("Practice makes perfect")
- Prepare well for exams
- Try to anticipate exam tasks

Weinhandl, R., Mayerhofer, M., Houghton, T., Lavicza, Z., Kleinferchner, L. M., Anđić, B., Eichmair, M., Hohenwarter, M.
Enhancing user-centred educational design: Developing personas of mathematics school students: Multimedia component 3.
Correspondence: Martin Mayerhofer, Department of Mathematics, Faculty of Mathematics, University of Vienna, Vienna, Austria, martin.mayerhofer@univie.ac.at

Persona prototype 5

Rather low level of performance

Tries to find a successful alternative to permanent studying

Hopes that being familiar with mathematical recipes will be sufficient

Uses additional resources as a support

Goals

- Pass grades in tests and in school reports
- Perform better in the next exam

Needs

- Materials and technological tools for illustration and support
- Sufficiently many explanations

Challenges & Problems

- When transferring internalised solution strategies to new tasks
- Connections not recognisable
- Organise help

Joys

- Hardly any
- When fixed schemes lead to the solution
- When receiving a pass grade

Fears

- Negative grades
- To fail in exams
- To ask classmates

Feelings & Emotions

- Nervousness
- Pressure, tension
- Blockade
- Frustration
- Respect for the subject

Strategies

- Memorise solution patterns, learn by heart
- Prepare for tests with diligence and tutoring
- Maybe ask classmates for help

Weinhandl, R., Mayerhofer, M., Houghton, T., Lavicza, Z., Kleinferchner, L. M., Anđić, B., Eichmair, M., Hohenwarter, M.
Enhancing user-centred educational design: Developing personas of mathematics school students: Multimedia component 3.
Correspondence: Martin Mayerhofer, Department of Mathematics, Faculty of Mathematics, University of Vienna, Vienna, Austria, martin.mayerhofer@univie.ac.at

Persona prototype 6

Passive

Indifferent & minimalistic

Would like to know the minimum requirements exactly

Goals

- Pass with as little effort as possible
- Finish work quickly

Needs

- Demonstrate indifference
- Be taken by the hand, to be guided
- Know exactly what to do
- Have sample tasks to fall back on
- Mathematical Recipes for achieving a pass grade

Challenges & Problems

- Problems trying to catch up on missed content on their own
- Does not see sense in learning mathematics
- Lack of motivation

Joys

- When finding the correct solutions
- Being able to recognise and apply clear rules and structures

Fears

- Huge effort necessary
- Fear of failure

Feelings & Emotions

- Indifferent, bored
- Despair
- Enjoyment in case of achieving a pass grade despite low effort
- Anger, rage

Strategies

- Try to find out minimum effort
- Test the limits
- Study for passing the examination, not for understanding mathematics
- Cheating
- Act passively, take things as they come
- Catch up on missed content

Weinhandl, R., Mayerhofer, M., Houghton, T., Lavicza, Z., Kleinferchner, L. M., Anđić, B., Eichmair, M., Hohenwarter, M.
Enhancing user-centred educational design: Developing personas of mathematics school students: Multimedia component 3.
Correspondence: Martin Mayerhofer, Department of Mathematics, Faculty of Mathematics, University of Vienna, Vienna, Austria, martin.mayerhofer@univie.ac.at

Persona prototype 7

Studies when there is a reason for studying
Starts studying when it is high time
Likes learning mathematical recipes
Believes they will not need mathematics in the future

Goals

- Pass the next exam, examination or assessment with an appropriate grade but without studying much

Needs

- Various opportunities for studying provided by the teacher or a private tutor
- Clear structures/procedures and recipes for solving exercises
- Clear instructions
- Sample solutions and exercises
- Getting help only when having a problem

Challenges & Problems

- Time management
- Establishing links between mathematical concepts and also between mathematics and everyday life
- Mathematics in non-standard contexts
- Motivation to study
- Organising help

Joys

- When you know a pattern that fits most of the time
- When a result is correct
- Repetitively solve exercises
- When they achieve the grade they have been aiming for

Fears

- New topics, technology or applications; wants to stay in a familiar environment
- Fail in exams

Feelings & Emotions

- Despair when new things are introduced
- Fear of failing in exams
- Stress when new topics are introduced and during preparation for exams
- Nervousness before exams
- Relief when an exam is over

Strategies

- Memorise patterns
- Study to become familiar with a topic just before an exam
- “Muddle through”, copy, and cheat in exams; is okay with any means of support to achieve a pass grade

Correspondence: Martin Mayerhofer, Department of Mathematics, Faculty of Mathematics, University of Vienna, Vienna, Austria, martin.mayerhofer@univie.ac.at

Persona prototype 8

Is good at mathematics and wants to deepen their knowledge

Deals with mathematical topics after school (either school-related topics or brain-twisters).

Is sometimes underchallenged

Wants to talk and discuss about mathematical topics and also inspire others

Goals

- Gain extensive mathematical knowledge
- Become familiar with mathematical reasoning
- Apply mathematics in other areas

Needs

- To talk with others about mathematics and applications
- To be challenged in class
- To understand connections (and receive explanations)
- To be able to demonstrate knowledge

Challenges & Problems

- When classes do not meet their needs; when they are underchallenged
- Sometimes calculations in class are done too fast or inaccurately
- When one has to simply accept something; when no explanation is given

Joys

- Discuss mathematics and applications
- Help others
- Identify mathematical connections (on their own)

Fears

- Not live up to expectations; present something wrong; that their solution might not be correct
- lose a reputation

Feelings & Emotions

- Proud of their own knowledge
- Disappointment when they do not receive answers to interesting questions

Strategies

- Use resources available at school (lessons, teachers, books) and draw upon them
- Extracurricular engagement with mathematics not covered at school or mathematical brain-twisters
- Active search for additional sources of knowledge

Weinhandl, R., Mayerhofer, M., Houghton, T., Lavicza, Z., Kleinferchner, L. M., Anđić, B., Eichmair, M., Hohenwarter, M.
Enhancing user-centred educational design: Developing personas of mathematics school students: Multimedia component 3.
Correspondence: Martin Mayerhofer, Department of Mathematics, Faculty of Mathematics, University of Vienna, Vienna, Austria, martin.mayerhofer@univie.ac.at

Persona prototype 9

Works and studies a lot for a good grade

Invests a lot of time, but rarely achieves outstanding results

Does not want teachers or classmates to see their weakness

Goals

- To achieve a pass grade (or do even better)
- Not be noticed and not be called on in class
- Wants to solve the given tasks and meet expectations

Needs

- Enough time and enough exercises for practice
- Wants to receive extensive and slow explanations
- Study without pressure or without having an examination in mind; without being watched by others

Challenges & Problems

- When materials are confusing or do not 100% familiar with the topic or lesson
- Making connections across mathematical areas
- When having to learn on their own; when having to find study materials on their own

Joys

- Study or work success (e.g., exam, school report) and get rewarded by others
- Solving exercises without help
- When they can study without the pressure of being graded

Fears

- Exams, school reports
- Not reaching a goal despite a lot of studying
- That they will be embarrassed in front of other people

Feelings & Emotions

- Respect for mathematics and people who are talented at mathematics
- Fear and respect: mathematics is important and difficult
- Anger when time investment does not lead to the desired result

Strategies

- Invest a lot of time in learning and also make use of external sources (private tutoring, internet)
- Fulfil all assignments (including voluntary tasks) given by the teacher
- Participate in class as much as possible

Correspondence: Martin Mayerhofer, Department of Mathematics, Faculty of Mathematics, University of Vienna, Vienna, Austria, martin.mayerhofer@univie.ac.at

Persona prototype 10

Well performing student who mainly studies for a good grade
Designs their own learning environment to achieve this goal

Goals

- To achieve a good grade (usually an A)
- To be able to solve all the tasks given to them

Needs

- To have a wide range of exercises given to them
- To be able to use different tools
- Works alone most of the time, unless help is needed
- Participates actively in lessons; the more if they realise that it is rewarded

Challenges & Problems

- Insecure when problems get complex or when they do not have a solution immediately at hand
- Impatience: they want to understand and solve things quickly
- Pressure when there are assessments on a highly specific topic

Joys

- Performing well in exams
- Positive feedback (especially from the teacher)
- Solving something in front of the class that improves recognition

Fears

- Not living up to expectations (not achieving a good grade)
- Lapsing in exams; not being able to perform when performing is demanded

Feelings & Emotions

- Impatience: wants to achieve goals quickly
- Enjoyment when getting a good grade
- Fear of failure or of not meeting expectations

Strategies

- Solve many exercises
- Prepare well for exams and for lessons
- Do things that might impress other people
- Use also external resources

Weinhandl, R., Mayerhofer, M., Houghton, T., Lavicza, Z., Kleinferchner, L. M., Anđić, B., Eichmair, M., Hohenwarter, M.
Enhancing user-centred educational design: Developing personas of mathematics school students: Multimedia component 3.
Correspondence: Martin Mayerhofer, Department of Mathematics, Faculty of Mathematics, University of Vienna, Vienna, Austria, martin.mayerhofer@univie.ac.at

Persona prototype 11

Average student who is okay with not performing well

No big problems in mathematics, also no ambitions to achieve more than what can be done easily

Goals

- To achieve an average grade with as little effort as possible

Needs

- To be told exactly what to do or to study
- Clear guidelines
- Being prescribed to follow a clear goal
- Being supplied with practice material

Challenges & Problems

- Motivating oneself (when no goal is set from someone else)
- Managing time to study for exams has become increasingly difficult
- Catching up on missed content has become a challenge

Joys

- When the exam is over; when having passed the exam
- When solving a task quickly

Fears

- Increasing workload
- Complex tasks or applications

Feelings & Emotions

- Emotionless and indifferent towards mathematics
- Enjoyment when having achieved something with little effort

Strategies

- Try to figure out what/how the exam will be
- Study to become familiar with a topic just before an exam
- Study as much as necessary to achieve a goal; even if things can be understood easily, they do not study more
- Aims at the best possible ratio of input to output
