# Supplementary material for: Enhancing user-centred educational design: Developing personas of mathematics school students
Source: Heliyon. 2024 Jan 7;10(2):e24173. doi: 10.1016/j.heliyon.2024.e24173 (PMC10827463; doi:10.1016/j.heliyon.2024.e24173)
Supplement: Multimedia component 4 [file mmc4.pdf]

Weinhandl, R., Mayerhofer, M., Houghton, T., Lavicza, Z., Kleinferchner, L. M., Anđić, B., Eichmair, M., Hohenwarter, M.

Enhancing user-centred educational design: Developing personas of mathematics school students

Multimedia component 4

Weinhandl, R., Mayerhofer, M., Houghton, T., Lavicza, Z., Kleinferchner, L. M., Anđić, B., Eichmair, M., Hohenwarter, M.
Enhancing user-centred educational design: Developing personas of mathematics school students: Multimedia component 4.
Correspondence: Martin Mayerhofer, Department of Mathematics, Faculty of Mathematics, University of Vienna, Vienna, Austria, martin.mayerhofer@univie.ac.at

Persona 1: Johannes Friedrich, 16 years

Johannes attends the 10th grade in a private grammar school. He is the eldest of three children and is often responsible for looking after his little brother and sister. His parents don't have a regular working schedule as project supervisor in the IT sector and as a self-employed podiatrist – therefore they make arrangements with their eldest son to take care of the other children from time to time. When there is free time for Johannes, he devotes himself to his interests in technology: He uses his self-taught programming skills to realise coding projects of increasing complexity. With additional materials and scientific magazines, he tries to compensate the needs the mathematics and science lessons at school fail to address. He hardly misses an opportunity to expand his mathematical knowledge and to understand complex relations. In mathematics, he is far ahead of his classmates and feels unchallenged by the speed at which things are taught and learnt in the lesson. This gives him the opportunity to successfully participate in mathematics competitions. Sometimes in mathematics lessons he supports the teacher by explaining some aspects once again for other pupils. Johannes is absolutely convinced that he will pursue studies in the field of mathematics and technology.

Goals

- Achieve the highest possible mathematical knowledge
- Understand concepts, reasons, and relations
- Apply mathematics in out-of-school contexts

Needs

- Discuss topics of and beyond school mathematics
- Be able to present mathematical knowledge and skills
- Resources to deepen and extend mathematical knowledge beyond what is taught in school
- Be challenged (also during lessons)

Challenges & Problems

- No opportunity for in-depth learning in class ("teaching to the test")
- Concepts have to be accepted – no line of reasoning is presented

Joys

- Learn something new when discussing about mathematics
- Solve complex problems successfully and draw connections
- Perceive and present their skills
- Support others

Fears

- Not live up to expectations/reputation, lose status
- Fail in front of the others
- Loss of enjoyment due to incomprehensible teaching

Feelings & Emotions

- Disappointment due to a lack of in-depth learning
- Enjoys maths
- Proud of his skills
- Appreciation of classmates

Strategies

- Self-initiated deepening
- Active search for external resources
- Active engagement with extracurricular material
- Teacher as a resource for learning

Weinhandl, R., Mayerhofer, M., Houghton, T., Lavicza, Z., Kleinferchner, L. M., Anđić, B., Eichmair, M., Hohenwarter, M.
Enhancing user-centred educational design: Developing personas of mathematics school students: Multimedia component 4.
Correspondence: Martin Mayerhofer, Department of Mathematics, Faculty of Mathematics, University of Vienna, Vienna, Austria, martin.mayerhofer@univie.ac.at

Persona 2: Aurelia Höfinger, 17 years

Aurelia attends the 11th grade at a grammar school but is entirely focused on her studies. She wants to study medicine and is already preparing for the entrance exam. In case she does not pass the entrance exam, she would like to study psychology for a year in order to prepare for another attempt. To avoid this extra year, she puts the preparation for the qualifying exam before anything else and wants to leave school with the best possible grades. She is aware of the fact that mathematics and technology play an important role in the entrance exam, which is also the reason for her interest in the subject. She works hard at school and tries to shine through additional tasks. Outside school she tries to preserve her skills in mathematics by repeatedly solving exercises to prevent lapsing in exams. Her parents and her sister, who is three years older and already studying at university, support Aurelia and her plan.

Goals

- (Very) Good grades
- Solve all exercises demanded by the teacher

Needs

- Have a wide range of exercises available
- Ask other people for support
- Positive feedback
- Get bonus points

Challenges & Problems

- Insecurity when it comes to fiddly tasks
- Impatience: everything should be understood and solved quickly
- Pressure in summative assessments on highly specific topics

Joys

- Positive feedback from people of authority
- Successful application of solution strategies
- (Very) Good grades

Fears

- Failing (lapsing) in summative assessments on highly specific topics

Feelings & Emotions

- Enjoys being rewarded
- Pressure to perform
- Impatience, time pressure

Strategies

- Put usefulness of mathematics in the foreground
- Solve many tasks, repetitively solve many exercises of prior exams
- Use external resources for more exercises
- Try to impress others and receive positive feedback

Weinhandl, R., Mayerhofer, M., Houghton, T., Lavicza, Z., Kleinferchner, L. M., Anđić, B., Eichmair, M., Hohenwarter, M.
Enhancing user-centred educational design: Developing personas of mathematics school students: Multimedia component 4.
Correspondence: Martin Mayerhofer, Department of Mathematics, Faculty of Mathematics, University of Vienna, Vienna, Austria, martin.mayerhofer@univie.ac.at

Persona 3: Manuel Winkler, 16 years

Manuel attends the 10th grade at a grammar school. He lives with his parents, a full-time employed shop assistant and a part-time employed aide. They support Manuel but consider him to be responsible for his school duties. He plans to go to university after school, therefore successful graduation is very important for him – he cares less about school itself and what has to be done for school. To successfully graduate, he is ready to reach for any strategy – regardless of being allowed or not – that helps him pass his exams and save time and effort. When preparing for a test, Manuel tries to anticipate the minimal amount of effort necessary to pass the exam. He likes best when his teacher specifies in detail what he needs to do to receive a positive grade. Up to the 8th grade he had been performing well in maths, afterwards the number of topics interesting for him decreased and his performance got worse. Once the situation forces him to raise his effort, he desires exercises that present rules and recipes that help him drill the relevant topics. All in all, Manuel approaches his tasks and lessons quite unemotionally and indifferently. He doesn't waste much thought, but is still happy when he achieves something with little effort. He is persistent when it comes to console games, which he spends hours on also at night.

Goals

- To reach a low goal with as little effort and time as possible

Needs

- To be told exactly what to do to
- Availability of mathematical recipes

Challenges & Problems

- Lack of motivation in the absence of a goal set by others
- Catching up on missed content in order to prepare for exams
- Time management
- Purpose/benefit of mathematics not recognisable – besides getting reasonable grades

Joys

- Being able to recognise and apply clear rules and structures in order to solve given tasks
- After an exam is done
- After passing an exam
- When goals are achieved easily

Fears

- Increased effort necessary to achieve the current goal
- Complex and time-consuming tasks

Feelings & Emotions

- Unemotional, indifferent
- Enjoys when something is achieved with little effort

Strategies

- Anticipate teacher expectations, try to find out minimum amount of effort
- Study to become familiar with a topic just before an exam
- Unallowed aids: copying, tricks with little effort

Weinhandl, R., Mayerhofer, M., Houghton, T., Lavicza, Z., Kleinferchner, L. M., Anđić, B., Eichmair, M., Hohenwarter, M.
Enhancing user-centred educational design: Developing personas of mathematics school students: Multimedia component 4.
Correspondence: Martin Mayerhofer, Department of Mathematics, Faculty of Mathematics, University of Vienna, Vienna, Austria, martin.mayerhofer@univie.ac.at

Persona 4: Diana Markovic, 15 years

Diana is attending the 9th grade at an upper secondary school and is thinking of changing to a school that prepares for a caring profession or commencing a caring profession. She moved to Austria with her parents when she was 7 years old. At school she considers some subjects very important and other subjects not important at all. For her, mathematics is one of the important subjects, although it is challenging for her. Her main goal is to receive positive grades, but she strives to perform better at the next opportunity. In her free time with her friends, she is very talkative and likes gossip that includes high society, while in class she is rather calm and does not like to stand out. Even when she is able to follow the teacher's explanations and to answer their questions, she usually keeps to herself and doesn't share her answers. When necessary, Diana receives private tutoring and searches for additional exercise materials that are of help for her. She has a patient nature and takes sufficient time to prepare for exams so that she can go up comfortably to her exams and to relieve her worries for not receiving satisfying grades.

Goals

- Pass grade or better grade than in the prior assessment
- To not attract attention
- To not be called on in class

Needs

- Study materials, technological aids to prepare for exams
- Sufficient time
- Detailed, slow explanations
- Be able to learn without pressure or having to perform, without being watched

Challenges & Problems

- Apply internalised concepts to new problems
- Study materials that do not fit her way of learning
- Organise help and supportive materials on her own

Joys

- In general little enjoyment
- Finding fixed schemes for solving tasks
- Achieving working goal
- Learning without the pressure of being graded

Fears

- Examinations
- Bad grades in spite of learning
- Embarrassing herself by asking for help

Feelings & Emotions

- Respect for the subject and for mathematically skilled people
- Fear, pressure, tension, nervousness
- Maths is regarded difficult and important
- Anger and frustration when the effort has not paid off

Strategies

- Invest a lot of time in internalising mathematical recipes
- Use study materials to practise
- Use a variety of sources (e.g., private tutoring, internet)

Weinhandl, R., Mayerhofer, M., Houghton, T., Lavicza, Z., Kleinferchner, L. M., Anđić, B., Eichmair, M., Hohenwarter, M.
Enhancing user-centred educational design: Developing personas of mathematics school students: Multimedia component 4.
Correspondence: Martin Mayerhofer, Department of Mathematics, Faculty of Mathematics, University of Vienna, Vienna, Austria, martin.mayerhofer@univie.ac.at

Persona 5: Marvin Beck, 15 years

Marvin attends the 9th grade at a grammar school. He is still unsure which way to go after school. He can imagine both working as a craftsman as well as studying at university with a technical orientation. His parents and his brother, who is three years younger, are pulling together with Marvin, support each other and are proud of their family. Marvin puts great stress on his appearance, always confidently applies gel to his hair and, since recent times, knocks his upper body into shape in a gym. His friends know well about his new sporting activity from Marvin's regular posts in social networks next to the updates about his successful appearances in his football team. His school achievements are average, especially maths poses a challenge for him, which he usually masters reasonably well. He wants to prove to his maths teacher, his colleagues, and his parents that he has some math skills down pat. Therefore, he works hard as an exam comes closer and tries to learn patterns and solutions by heart, sometimes supported by a private tutor. He gets nervous when he thinks about the possibility to perform worse than people that are important to him would expect.

Goals

- Pass exams with a satisfying grade
- Complete homework reasonably well

Needs

- Many resources provided for studying
- Clear guidelines and ways of solving problems
- Sample answers, mathematical recipes to internalise
- Possibilities to illustrate things
- Quick help within reach at all times

Challenges & Problems

- Mathematics appearing in unfamiliar contexts
- Time management
- Organising help
- Motivating himself
- Tasks that are structured differently than he is used to

Joys

- When a pattern is found that frequently leads to success
- When an internalised strategy helps to solve exercises
- Pass grades

Fears

- Presenting wrong results
- Failing in exams
- New topics that cannot be connected easily to familiar ones

Feelings & Emotions

- Prejudiced against and desperate towards new topics
- Relieved when having passed exams
- Not sure if the time invested for exam preparation would be enough → nervousness before exams

Strategies

- Memorise patterns and solutions
- Prepare for exams just on time
- Tutoring or studying with friends before exams
- Use any promising means (e.g., copying, cheating)
