# Supplementary material for: Enhancing user-centred educational design: Developing personas of mathematics school students
Source: Heliyon. 2024 Jan 7;10(2):e24173. doi: 10.1016/j.heliyon.2024.e24173 (PMC10827463; doi:10.1016/j.heliyon.2024.e24173)
Supplement: Multimedia component 5 [file mmc5.pdf]

Weinhandl, R., Mayerhofer, M., Houghton, T., Lavicza, Z., Kleinferchner, L. M., Anđić, B., Eichmair, M., Hohenwarter, M.

Enhancing user-centred educational design: Developing personas of mathematics school students

Multimedia component 5

English version

Weinhandl, R., Mayerhofer, M., Houghton, T., Lavicza, Z., Kleinferchner, L. M., Anđić, B., Eichmair, M., Hohenwarter, M.
Enhancing user-centred educational design: Developing personas of mathematics school students: Multimedia component 5.
Correspondence: Martin Mayerhofer, Department of Mathematics, Faculty of Mathematics, University of Vienna, Vienna, Austria, martin.mayerhofer@univie.ac.at

Johannes Friedrich

16 years

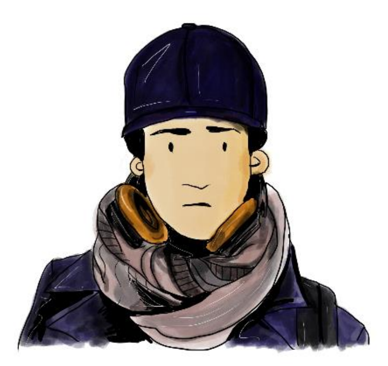

Johannes is in the 10th grade at a private preparatory school. His parents, a nurse and a project supervisor in the IT sector, don't have a regular working schedule. Johannes is an only child and takes on household chores when his parents are at work. His parents expect him to finish his homework on his own in a responsible way. In his spare time, Johannes has several hobbies related to science and engineering: He has picked up basic coding skills by himself and uses them to work on projects of increasing complexity. Johannes frequently uses sources such as science magazines that go above and beyond what he is taught in school. He hardly misses an opportunity to learn about math. Johannes is far ahead in maths and usually bored in class. Johannes uses the time gained to support the teacher by helping students who lag behind or by working on challenging problems to prepare for national maths competitions in which he is doing well. Johannes has already made up his mind that he will pursue studies in the field of mathematics, science, or engineering at university.

Goals

- As much knowledge in maths as possible
- Understanding of mathematical concepts
- Applying maths in out-of-school and interdisciplinary contexts

Needs

- Opportunities to discuss maths, also beyond the curriculum
- Opportunities to show his math skills
- Access to materials that go beyond the curriculum
- Extra challenges in and outside of school

Challenges & Problems

- Lack of opportunities in class to go beyond the curriculum
- Presentation of new mathematical concepts with little or no proof in class

Joys

- Learn something new when discussing math
- Solve challenging problems
- Discover connections between different problems or areas
- Feel his mathematical prowess and to show it off
- Help others

Fears

- Fall short of others' expectations
- Lose his status
- Fail publicly
- Lose interest in mathematics on account of superficial lessons

Feelings & Emotions

- Enjoying math
- Disappointment by superficial presentations of concepts
- Pride in his skills
- Appreciation by peers and teachers

Strategies

- Independent study
- Actively searches for additional resources
- Engages in extracurricular activities related to maths
- Approaches his teachers with advanced questions after class

Correspondence: Martin Mayerhofer, Department of Mathematics, Faculty of Mathematics, University of Vienna, Vienna, Austria, martin.mayerhofer@univie.ac.at

Aurelia Höfinger

17 years

Aurelia is in the 11th grade at a preparatory school. Her focus is already on studying medicine. She has already started to prepare for the entrance exam to study medicine. If she doesn't pass at the first go, she plans to enrol for psychology for one year and then retake the entrance exam. Her goal, however, is to pass at her first attempt and also to finish school with straight A's. She is aware of the fact that maths play an important role in the exam, which explains her interest in the subject. Aurelia works hard at school and attempts to stand out by doing extra work. Outside school she tries to hone her math skills by solving the same exercises over and over again to prevent lapsing in an exam.

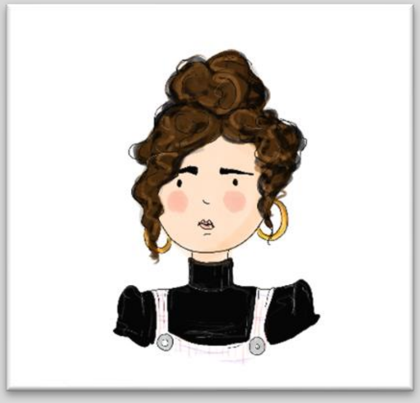

Aurelia's parents and her sister, who is three years older and already studying at university, are supportive of Aurelia and her plan.

Goals

- Straight A's
- Solving all exercises requested by teachers

Needs

- Large supply of practice problems
- Additional support from people other than the teachers
- Reassurance
- Extra credit

Challenges & Problems

- Impatience with open, unclear, tricky, or unfamiliar problems
- Stress in exams

Joys

- Receive positive feedback from her teachers
- Successfully apply familiar strategies
- Excel in exams

Fears

- Not excel in exams
- Have a mental block in exams

Feelings & Emotions

- Thrill when being rewarded
- Pressure to perform well
- Impatience

Strategies

- Treats maths as a means to her future studies
- Practises the same problems many times over
- Uses external sources to find additional practice problems
- Elicits positive feedback from her teachers

Weinhandl, R., Mayerhofer, M., Houghton, T., Lavicza, Z., Kleinferchner, L. M., Anđić, B., Eichmair, M., Hohenwarter, M.
Enhancing user-centred educational design: Developing personas of mathematics school students: Multimedia component 5.
Correspondence: Martin Mayerhofer, Department of Mathematics, Faculty of Mathematics, University of Vienna, Vienna, Austria, martin.mayerhofer@univie.ac.at

Manuel Winkler

16 years

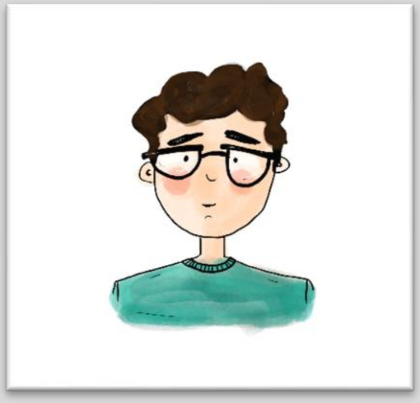

Manuel is in the 10th grade at a preparatory school. He lives with his parents, a full-time shop assistant and a cleaning person working part-time. They consider Manuel to be responsible for his school work and are generally supportive. He plans to go to university after school. Finishing high school is what matters most to Manuel right now. He is not willing to put in more effort into school than absolutely necessary. Manuel is willing to adopt *any* strategy – permitted or not – that helps him pass his exams and save time and effort. He tries to anticipate the minimal amount of effort necessary to pass exams. Manuel wants his teachers to specify in detail what needs to be done to pass the class. Up to 8th grade, Manuel did very well at maths. Since then the number of topics that interest him in maths class have decreased and his performance got poorer. Manuel looks for materials that present rules and recipes that help him cram for exams. Overall, Manuel approaches his school work unemotionally and quite indifferently. He prefers to play video games for many hours on end. He doesn't spend much thought on school-related issues. Manuel is pleased when he passes exams or tasks with as little effort as possible.

Goals

- Passing with as little effort and commitment as necessary

Needs

- Needs to know exactly the passing requirements
- Mathematical recipes

Challenges & Problems

- Lack of motivation unless his teachers or parents set goals
- Cramming just before the exam
- No incentive to study maths other than to pass

Joys

- Apply rules that are easy to understand and help him solve the required tasks
- Pass exams no matter the grade
- Reach his goals with the least amount of time and effort

Fears

- Having to put in more effort to pass
- Having to work on exercises that cannot be solved following a familiar recipe or rule

Feelings & Emotions

- Indifference

Strategies

- Tries to anticipate exam questions
- Tries to find out exactly the passing requirements
- Prepares for exams just in time
- Cheats

Correspondence: Martin Mayerhofer, Department of Mathematics, Faculty of Mathematics, University of Vienna, Vienna, Austria, martin.mayerhofer@univie.ac.at

Diana Markovic

15 years

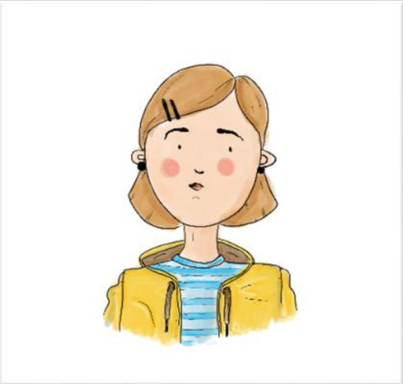

Diana is in the 9th grade at an upper secondary school specialised on STEM subjects and is thinking of quitting school and taking up training as a caregiver or transferring to school that specialises in social work. She moved to Austria with her parents at the age of seven. At school, Diana considers some subjects very important and others as not important at all. She considers maths important, even though she faces challenges. Her main goal is to pass, however, she always wishes to do better in the future. Compared to her sociable and talkative nature when she is with her friends, she is rather quiet and doesn't want to draw attention in class. Even when she is following well, she doesn't usually volunteer her answer or ideas to questions the teacher asks to the class. Occasionally, Diana receives private tutoring and uses additional outside materials to help her study. She is diligent and takes sufficient time to prepare for exams, so she can be relaxed before exams and doesn't have to worry about poor grades.

Goals

- Pass and do better next time
- Not be called on by teachers in class

Needs

- Additional study materials
- Visualisations for concepts
- Sufficient time to prepare for exams
- Step-by-step explanations

Challenges & Problems

- Applying acquired skills in unfamiliar contexts
- Having to use materials that do not fit her visual learning style
- Organising help and additional materials by herself

Joys

- Little joy in maths
- Solve routine problems
- Study without pressure

Fears

- Test anxiety
- Causing embarrassment by asking for help on supposedly easy problems

Feelings & Emotions

- Respect for maths and for people who are good at maths
- Anxiety and pressure
- Considers maths difficult and important
- Frustration when her efforts do not pay off

Strategies

- Plans ahead to have sufficient time for studying
- Uses additional materials to prepare for exams
- Uses multiple means of support (private tutoring, internet resources, etc.)

Correspondence: Martin Mayerhofer, Department of Mathematics, Faculty of Mathematics, University of Vienna, Vienna, Austria, martin.mayerhofer@univie.ac.at

Dominik Ghali

15 years

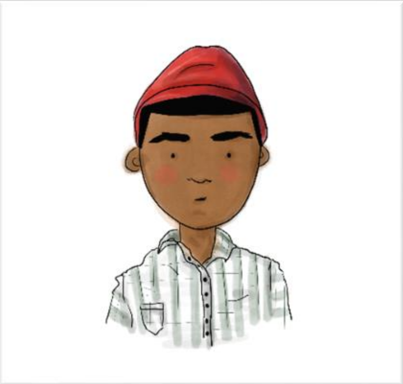

Dominik is in the 9th grade at a preparatory school. He has not yet decided what to do after finishing high school: He has considered both a career as a craftsman or to study for an engineering degree at university. His parents and his brother, who is three years younger, support Dominik and are proud of their family. Dominik pays much attention to his appearance, confidently applying gel to his hair and, as of recently, works out at a gym. He keeps his friends up to date on his progress in the gym and his appearances on his football team by regular posts in social networks.

His performance in school is average. Maths is especially challenging for Dominik, which he usually masters quite well. He wants to prove to his maths teacher, his colleagues, and his parents that he has talent for maths. Therefore he works hard as exams draw closer and commits solutions to practise problems to heart, sometimes assisted by a private tutor. Dominik is anxious at the thought of performing worse than people who are important to him would expect.

Goals

- Passing exams with grades that reflect the effort he has put in
- Doing a reasonable job on his homework

Needs

- Large amount of study materials
- Clear understanding of what is required to get a reasonable grade
- Model solutions and problem solving strategies
- Visualisations of concepts
- Additional support within reach at all times

Challenges & Problems

- New and unfamiliar contexts
- Time management
- Organising support on his own
- Motivating himself to study
- Tasks that are structured differently from what he is used to

Joys

- Finding a pattern that frequently leads to a solution
- Solving an exercise using an internalised strategy
- Positive grades

Fears

- Fail in exams
- Fail to connect with completely new topics

Feelings & Emotions

- Intimidated by new concepts
- Relieved when passing exams
- Not confident that effort will pay off
- Anxious before exams
- Resilience

Strategies

- Commit solutions of practice problems and solution patterns to heart
- Prepare for exams just enough
- Prepare for exams together with friends or a private tutor
- Use *any* means promising a better result (also cheating)

Weinhandl, R., Mayerhofer, M., Houghton, T., Lavicza, Z., Kleinferchner, L. M., Anđić, B., Eichmair, M., Hohenwarter, M.

Enhancing user-centred educational design: Developing personas of mathematics school students

Multimedia component 5

German version (original)

Weinhandl, R., Mayerhofer, M., Houghton, T., Lavicza, Z., Kleinferchner, L. M., Anđić, B., Eichmair, M., Hohenwarter, M.

Correspondence: Martin Mayerhofer, Department of Mathematics, Faculty of Mathematics, University of Vienna, Vienna, Austria, martin.mayerhofer@univie.ac.at

Johannes Friedrich

16 Jahre

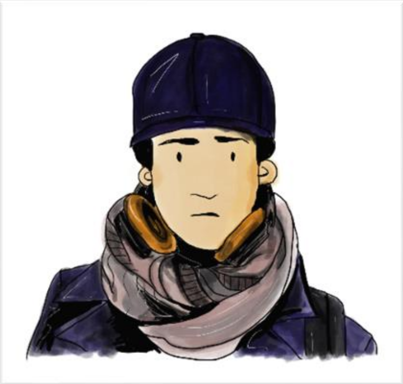

Johannes besucht ein privates Gymnasium und geht dort in die 6. Klasse. Seine Eltern haben als Projektbetreuer in einer IT-Firma bzw. als Selbstständige in der mobilen Fußpflege mitunter unregelmäßige Arbeitszeiten. Sie haben mit Johannes, ihrem einzigen Kind, vereinbart, welche kleineren Tätigkeiten er im Haushalt übernimmt, wenn beide Eltern in der Arbeit sind, und erwarten, dass er seine Aufgaben für die Schule selbstständig und verlässlich erledigt. Wenn Johannes frei über seine Freizeit verfügen kann, geht er seinen Interessen im Bereich der Technik nach: Er hat sich selbst Programmieren beigebracht und setzt immer komplexere Projekte um. Mit zusätzlichen Materialien und einem Abo eines naturwissenschaftlichen Magazins versucht er das zu bekommen, was ihm der Mathematik- und der naturwissenschaftliche Unterricht nicht bieten können. Er lässt wenig Gelegenheiten aus, in denen er sein mathematisches Wissen erweitern und komplexere Zusammenhänge verstehen versucht. In Mathematik ist er seinen Klassenkolleg*innen weit voraus und mit dem Unterrichtstempo unterfordert, was ihm die Gelegenheit gibt, an Mathematik-Wettbewerben teilzunehmen und dort erfolgreich abzuschneiden. Hin und wieder unterstützt er auch die Lehrperson, indem er für andere Schüler eine Sache nochmal erklärt. Für Johannes ist schon jetzt klar, dass er ein mathematisch-technisches Studium angehen möchte.

Ziele

- Möglichst umfangreiches mathematisches Wissen
- Konzepte sowie Begründungen und Zusammenhänge verstehen
- Mathematik in außermathematischen/ außerschulischen Kontexten anwenden

Bedürfnisse

- Sich über schulmathematische und außer-schulmathematische Themen austauschen
- Mathematisches Wissen und Können präsentieren können
- Ressourcen zur Vertiefung und Erweiterung der Schulmathematik
- Gefordert werden

Herausforderungen & Probleme

- Kein Raum für Vertiefung im Unterricht (teaching to the test)
- Konzepte müssen hingenommen oder selbst vertieft werden – im Unterricht gibt es keine Begründungen hierfür

Freude

- Über Mathematik diskutieren und dabei dazulernen
- Erfolgreiches Lösen komplexer Aufgaben und Herstellen von Zusammenhängen
- Wahrnehmung und Präsentation der eigenen Kompetenz
- Anderen helfen

Angst

- Erwartungen/Ruf nicht gerecht warden
- Status verlieren
- Öffentlich scheitern
- Verlust der Freude am Fach wegen unverständlichen Unterrichts

Gefühle & Emotionen

- Enttäuschung über mangelnde Vertiefung
- Freude am Fach
- Stolz auf eigenes Können
- Wertschätzung in der "Community"

Strategien

- Selbstständige Vertiefung
- Aktive Suche nach externen Ressourcen
- Aktive Beschäftigung mit außerschulischen Inhalten
- Stellt der Lehrperson außerhalb des Unterrichts vertiefende Fragen

Weinhandl, R., Mayerhofer, M., Houghton, T., Lavicza, Z., Kleinferchner, L. M., Anđić, B., Eichmair, M., Hohenwarter, M.
Enhancing user-centred educational design: Developing personas of mathematics school students: Multimedia component 5.
Correspondence: Martin Mayerhofer, Department of Mathematics, Faculty of Mathematics, University of Vienna, Vienna, Austria, martin.mayerhofer@univie.ac.at

Aurelia Höfinger

17 years

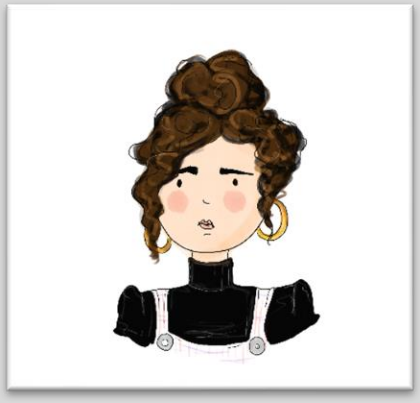

Aurelia besucht die 7. Klasse an einem Gymnasium, ist jedoch schon völlig auf das Studium fokussiert. Sie möchte Medizin studieren und bereitet sich schon in dieser Phase auf die Aufnahmeprüfung vor. Falls sie die Aufnahmeprüfung nicht schafft, möchte sie für ein Jahr Psychologie studieren, um schließlich einen weiteren Versuch für den Medizin-Aufnahmetest zu starten und sich mit diesem Übergangsjahr auch in gewisser Weise auf den Test vorzubereiten. Damit dieses Jahr nicht notwendig wird, ordnet sie dem Bestehen der Aufnahmeprüfung alles unter und möchte die Schule mit möglichst guten Noten abschließen. Dass Mathematik und Technik bei der Aufnahmeprüfung eine wichtige Rolle spielt, ist ihr bewusst, worauf auch ihr Interesse für das Fach zurückzuführen ist. Sie ist im Unterricht sehr bemüht und versucht, durch zusätzliche Leistungen positiv aufzufallen. Außerhalb des Unterrichts versucht sie sich durch wiederholtes Lösen von Prüfungsaufgaben für Leistungsüberprüfungen fit zu machen, um ein Blackout bei einer Prüfung zu vermeiden. Ihre Eltern und ihre drei Jahre ältere Schwester, die bereits im Studium steht, unterstützen Aurelias Plan.

Ziele

- (Sehr) Gute Note
- Alle vorgegebenen Aufgabenstellungen lösen

Bedürfnisse

- Große Auswahl an Übungsaufgaben vorgeschlagen bekommen
- Andere Personen zur Unterstützung heranziehen
- Positive Rückmeldungen
- Pluspunkte sammeln

Herausforderungen & Probleme

- Verunsicherung bei Aufgaben, bei denen man tüfteln muss
- Ungeduld: alles soll klar zu verstehen und zu lösen sein
- Druck bei punktuellen Überprüfungen

Freude

- Positive Rückmeldung von Autoritätspersonen
- Erfolgreiches Anwenden von Lösungsstrategien
- (Sehr) Gute Note

Angst

- Bei punktuellen Überprüfungen nicht erfolgreich sein (Blackout)

Gefühle & Emotionen

- Freude an Belohnungen
- Leistungsdruck
- Ungeduld, Zeitdruck

Strategien

- Zweckmäßigkeit von Mathematik in den Vordergrund stellen
- Viele Beispiele lösen, repetitives Lösen von vielen Prüfungsbeispielen
- Externe Ressourcen für mehr Übungsmaterialien heranziehen
- ndere Personen beeindrucken und positive Rückmeldungen herbeiführen versuchen

Weinhandl, R., Mayerhofer, M., Houghton, T., Lavicza, Z., Kleinferchner, L. M., Anđić, B., Eichmair, M., Hohenwarter, M.
Enhancing user-centred educational design: Developing personas of mathematics school students: Multimedia component 5.
Correspondence: Martin Mayerhofer, Department of Mathematics, Faculty of Mathematics, University of Vienna, Vienna, Austria, martin.mayerhofer@univie.ac.at

Manuel Winkler

16 Jahre

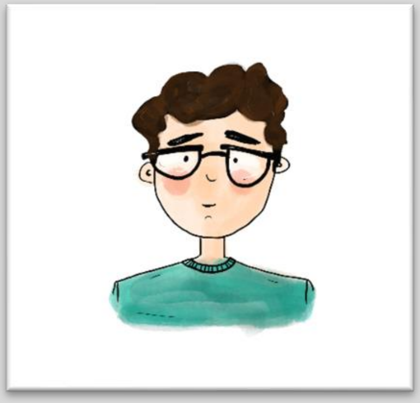

Manuel besucht die 6. Klasse eines Gymnasiums. Er lebt zusammen mit seinen Eltern, die Vollzeit im Handel bzw. Teilzeit als Hilfskraft berufstätig sind. Diese kümmern sich um Manuel, lassen ihn in schulischen Angelegenheiten jedoch selbst walten. Der Schulabschluss ist für ihn wichtig, weil er später an einer Universität studieren möchte, die Schule selbst und das, was dafür zu erledigen ist, jedoch weniger. Für das Erreichen des Schulabschlusses wählt er den Weg des geringsten Widerstandes und möchte mit möglichst wenig Anstrengung durchkommen. Deshalb ist ihm auch jedes erlaubte und unerlaubte Mittel recht, das ihm eine positive Note auf Leistungsfeststellungen beschert und keinen Aufwand macht. In der Vorbereitung auf Schularbeiten versucht er genau auf den Punkt hinzulernen und zu antizipieren, was für eine positive Note notwendig sein wird. Am liebsten ist ihm, wenn er genau vorgegeben bekommt, was zu tun ist und welche Aktivitäten er für eine positive Note setzen muss. In Mathematik war er bis gegen Ende der Unterstufe richtig gut, danach sind die Themen, die ihn interessieren, und auch seine Leistungen immer weniger geworden. Wenn ihn einmal die Situation dazu zwingt, aktiver zu werden, dann wünscht er sich für Mathematik Übungsmaterialien, die ihm Regeln und Rezepte präsentieren, mit denen er Inhalte rasch einüben kann. Insgesamt geht Manuel sehr emotionslos und gleichgültig an die Sache heran und verschwendet möglichst wenig Gedanken, freut sich aber, wenn er etwas mit geringem Aufwand geschafft hat. Ausdauernd zeigt er sich bei Computerspielen, denen er sich auch nachts stundenlang widmet.

Ziele

- Passing with as little effort and commitment as necessary

Bedürfnisse

- genau vorgegeben bekommen, was zu tun ist (wenn...dann…)
- Verfügbarkeit von Rezepten

Herausforderungen & Probleme

- fehlende Motivation, wenn ein fremdbestimmtes Ziel fehlt
- nachlernen vor Prüfungen
- Zeiteinteilung
- Sinnhaftigkeit/Nutzen über Note hinaus nicht erkennbar

Freude

- klare Regeln und Strukturen zum Lösen vorgegebener Aufgaben erkennen und anwenden können
- nach erledigten Prüfungen
- nach bestandenen Prüfungen
- bei schneller Zielerreichung

Angst

- Aufwand wird immer mehr, um aktuelles Ziel zu erreichen
- Beispiele, die nicht nach Rezept bearbeitet werden können

Gefühle & Emotionen

- emotionslos, gleichgültig
- Freude, wenn etwas mit geringem Aufwand geschafft wurde

Strategien

- Lehrer lesen, Mindestaufwand herausfinden versuchen
- auf den Punkt hinlernen
- unerlaubte Hilfsmittel: Abschreiben, Tricksereien mit wenig Aufwand

Weinhandl, R., Mayerhofer, M., Houghton, T., Lavicza, Z., Kleinferchner, L. M., Anđić, B., Eichmair, M., Hohenwarter, M.
Enhancing user-centred educational design: Developing personas of mathematics school students: Multimedia component 5.
Correspondence: Martin Mayerhofer, Department of Mathematics, Faculty of Mathematics, University of Vienna, Vienna, Austria, martin.mayerhofer@univie.ac.at

Diana Markovic

15 Jahre

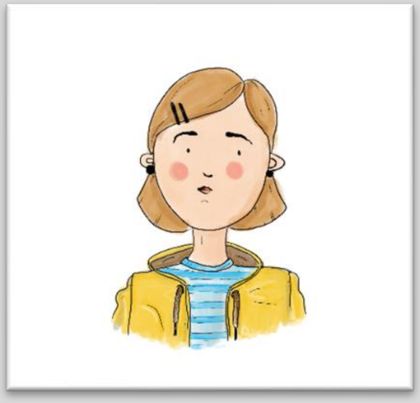

Diana besucht derzeit die 5. Klasse an einem ORG und überlegt in eine weiterführende Schule mit Schwerpunkt auf Sozialberufen oder einen Sozialberuf zu wechseln. Sie ist mit ihren Eltern nach Österreich gezogen, als sie 7 Jahre alt war. In der Schule gibt es Fächer, die sie sehr und andere, die sie gar nicht wichtig findet. Mathematik gehört für sie zu den wichtigen Fächern, allerdings stellt dieses Fach für sie eine Herausforderung dar. Ihr primäres Ziel ist es, eine positive Note zu erhalten, strebt nach einer Prüfung jedoch immer an, bei der nächsten besser abzuschneiden. Im Vergleich zur Freizeit mit ihren Freundinnen, in der sie sehr gesprächig ist und gerne über die High Society tratscht, ist sie im Unterricht zurückhaltend und möchte nicht auffallen. Auch wenn sie dem Unterricht folgen kann und eine richtige Antwort weiß, meldet sie sich nicht zu Wort. Als Unterstützung für das Lernen bezieht sie bei Bedarf Nachhilfe und sucht an vielen Stellen nach Materialien, die ihr weiterhelfen. Sie ist geduldig und nimmt sich viel Zeit für die Prüfungsvorbereitung, damit sie beruhigter in eine Prüfung gehen kann und ihre Sorge, dass sie trotz Lernens eine nicht zufriedenstellende Note bekommt, gelindert wird.

Ziele

- positive, bessere Note
- nicht auffallen
- nicht drangenommen werden

Bedürfnisse

- Lernmaterialien, technologische Hilfsmittel zur Prüfungsvorbereitung
- genügend Zeit
- umfangreiche, langsame Erklärungen
- ohne Druck/Prüfung, ohne gesehen zu werden lernen können

Herausforderungen & Probleme

- antrainierte Konzepte auf neuartige Probleme anwenden
- Lernmaterialien, die nicht zum eigenen Lernschema passen
- Hilfe organisieren, sich selbst Hilfsmaterialien organisieren

Freude

- grundsätzlich wenig Freude, wenn dann:
- fixe Schemata zum Lösen von Aufgaben gefunden
- Arbeitsziel erreicht
- ohne Notendruck lernen

Angst

- vor Leistungsüberprüfungen
- schlechte Note trotz Lernens
- sich durch Herbeiziehen von Hilfe blamieren

Gefühle & Emotionen

- Respekt und Ehrfurcht vor dem Fach und guten Personen
- Angst, Druck, Anspannung, Nervosität
- Mathematik ist schwierig und wichtig
- Ärger und Frust, wenn Investitionen nicht reichen

Strategien

- Viel Zeit in das (Rezept-)Lernen investieren
- mit Lernmaterialien üben
- vielfältige Unterstützung und Quellen heranziehen (Nachhilfe, Internet, …)

Weinhandl, R., Mayerhofer, M., Houghton, T., Lavicza, Z., Kleinferchner, L. M., Anđić, B., Eichmair, M., Hohenwarter, M.
Enhancing user-centred educational design: Developing personas of mathematics school students: Multimedia component 5.
Correspondence: Martin Mayerhofer, Department of Mathematics, Faculty of Mathematics, University of Vienna, Vienna, Austria, martin.mayerhofer@univie.ac.at

Dominik Ghali

15 Jahre

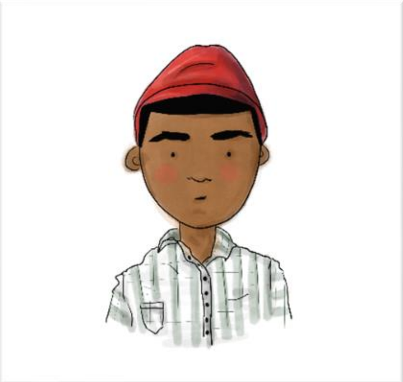

Dominik geht in die 5. Klasse an einem Gymnasium. Er ist noch unsicher, wie seine Laufbahn weitergehen wird, und kann sich nach der Schule derzeit sowohl einen technisch-handwerklichen Beruf als auch eine tertiäre Ausbildung mit technischer Orientierung vorstellen. Seine Eltern und sein drei Jahre jüngerer Bruder ziehen mit Dominik an einem Strang, geben sich gegenseitig Rückhalt und sind stolz auf ihre Familie. Dominik legt wert auf sein Erscheinungsbild, gelt seine Haare immer stilsicher hoch und trainiert seit Kurzem seinen Oberkörper im Fitnessstudio, was er auch seine Gefolgschaft in den Sozialen Netzwerken wissen lässt. Nach wie vor nimmt man auch regelmäßig Beiträge von ihm über seine fußballerischen Aktivitäten und Erfolge wahr.

Seine schulischen Leistungen sind durchschnittlich, insbesondere Mathematik stellt für ihn eine Herausforderung dar, die er aber meist passabel meistert. Bei Schularbeiten möchte er seiner Lehrperson, seinen Mitschüler*innen und seinen Eltern beweisen, dass er etwas kann, deswegen wird er im Vorfeld der Schularbeiten sehr aktiv und versucht Muster und Lösungswege auch mit Nachhilfe auswendig zu lernen. Nervosität bereitet ihm der Gedanke, dass seine Leistung schlechter ausfällt als die Menschen, die ihm wichtig sind, erwarten würden.

Ziele

- Prüfungen mit einer zufriedenstellenden Note absolvieren
- Hausübungen passabel erledigen

Bedürfnisse

- viele Lernangebote, die bereitgestellt werden
- Klare Vorgaben und Lösungswege
- Musterlösungen, Rezepte verinnerlichen
- Möglichkeiten zur einfachen Veranschaulichung
- schnelle Hilfe immer in Griffweite

Herausforderungen & Probleme

- Auftreten von Mathematik in ungewohnten, unbekannten Kontexten
- Zeiteinteilung
- sich Hilfe organisieren
- sich motivieren
- Materialien, die anders aufgebaut sind als das gedankliche Gerüst zu einem Thema

Freude

- wenn ein Muster gefunden wurde, das meistens zum Erfolg führt
- wenn eine antrainierte Strategie zum Lösen von Aufgaben führt
- an positiven Noten

Angst

- Prüfungen nicht bestehen
- vor neuen Themen, bei denen sich nicht leicht eine Verbindung zu Bekanntem herstellen lässt

Gefühle & Emotionen

- Voreingenommenheit gegenüber und Verzweiflung bei neuen Themen
- Erleichterung bei erledigten Prüfungen
- Unsicherheit, ob Investitionen reichen → Nervosität vor Prüfungen
- kann Rückschläge gut wegstecken

Strategien

- Muster und Lösungswege auswendig lernen
- sich punktgenau auf Prüfungen vorbereiten
- Nachhilfe oder mit Freunden vor Prüfungen lernen
- jedes erfolgversprechende Mittel verwenden (Abschreiben, Schummeln, …)
