# Supplementary material for: Enhancing user-centred educational design: Developing personas of mathematics school students
Source: Heliyon. 2024 Jan 7;10(2):e24173. doi: 10.1016/j.heliyon.2024.e24173 (PMC10827463; doi:10.1016/j.heliyon.2024.e24173)
Supplement: Multimedia component 6 [file mmc6.pdf]

Weinhandl, R., Mayerhofer, M., Houghton, T., Lavicza, Z., Kleinferchner, L. M., Anđić, B., Eichmair, M., Hohenwarter, M.

Enhancing user-centred educational design: Developing personas of mathematics school students

Multimedia component 6

Correspondence: Martin Mayerhofer, Department of Mathematics, Faculty of Mathematics, University of Vienna, Vienna, Austria, martin.mayerhofer@univie.ac.at

Classification of themes into persona categories

Goals

- Understanding mathematics
- Orientation of goals to own performance reference standard
- Effort avoidance

Needs

- Have a stress and pressure free learning environment
- Availability of materials (resources)
- Contact persons (resources)
- Have recipes available for solving mathematical problems

Challenges and problems

- Pressure situations, especially during exams
- Non-standard or in-depth tasks
- Time and resource management
- Lack of motivation
- Monotonous classes

Enjoyments

- Being cognitively challenged
- Presenting mathematical knowledge
- Receiving praise and rewards
- Finding and using patterns to solve problems
- Little time/resources investment

Fears

- Losing status
- Being embarrassed in front of others
- Fail in exams
- Having to invest more resources

Feelings and emotions

- Joy
- Nervous
- Being impressed/insecure/intimidated
- Frustrated
- Indifferent

Strategies

- Independent, active learning
- Investment of time/resources
- Repeated solving of tasks and memorising
- Use of external resources
- Copying and cheating
- Anticipating teacher actions
